# Utilization of a High-Pressure Vibrating Tube Densimeter for Liquids at Temperatures Down to 100 K

**DOI:** 10.1007/s10765-024-03357-9

**Published:** 2024-04-19

**Authors:** Nils von Preetzmann, Daniel Zipplies, Roland Span, Markus Richter

**Affiliations:** 1 Thermodynamics, Ruhr University Bochum, 44780 Bochum, Germany; 2 Applied Thermodynamics, Chemnitz University of Technology, 09107 Chemnitz, Germany

**Keywords:** Cryogenic state, Density measurement, Extended calibration, Feasibility study, Vibrating tube densimeter

## Abstract

A high-pressure vibrating tube densimeter, specified by the manufacturer for temperatures from (263 to 473) K at pressures up to 140 MPa, was tested at temperatures down to 100 K and from vacuum to pressures up to 10 MPa. To verify the functionality and overall performance under these conditions, the densimeter was calibrated with measurements under vacuum as well as methane and propane as reference fluids. The calibration range is *T* = (120 to 200) K at pressures from (2.0 to 10.0) MPa. To evaluate the recorded data, two established calibration models were used to describe the dependence of the densimeter's oscillation period on the investigated reference fluids' temperature, pressure, and density. The experiments showed that the vibrating tube densimeter is operational even at temperatures down to 100 K, but exhibits a shift of its vacuum resonance when subjected to thermal cycling at temperatures below 180 K. Accordingly, the calibration models were modified with respect to how the vacuum resonance is considered. Then, the determined calibration parameters reproduce the densities of the reference fluids within ± 0.10 kg·m^−3^ for the calibration model that performed better for the present study. Measurements on pure ethane and argon validate the calibration of the densimeter. Here, the densities are within (− 0.47 to 0.16) kg·m^−3^ of values calculated with the respective reference equation of state. The estimated combined expanded uncertainty (*k* = 2) in density for the validation measurements ranges from (0.52 to 1.13) kg·m^−3^ or is less than 0.1 % for liquid densities.

**Supplementary Information:**

The online version of this article (10.1007/s10765-024-03357-9) contains supplementary material, which is available to authorized users.

## Introduction

Accurate knowledge of the densities of compressed fluids is relevant for various technical and scientific applications. For the prediction of thermodynamic properties such as density, fundamental equations of state (EOS) or empirical correlations are used to reflect their dependencies on, for example, temperature and pressure. Comprehensive experimental datasets are required for the development and improvement of such models. Various established measuring methods are available for fluid density [1, 2]. State-of-the-art precision densimeters are often based on the Archimedes principle, employing one or more buoyancy sinkers in conjunction with magnetic suspension balances [3, 4], leading to complex densimeter designs and comprehensive control technologies. On the other hand, vibrating tube densimeters (VTDs) allow reasonably low system complexity while providing fast measurements with still very good uncertainties [5, 6], especially when considering high liquid densities. Commercially available VTDs enable fast and robust operation with considerably lower planning and acquisition costs than a gravimetric densimeter.

In general, VTDs are used primarily at elevated temperatures of up to 723 K [6], for example, for process monitoring or quality assurance along production chains. Considering low temperatures well below ambient temperature, vibrating tube densimeters have so far only been used to a limited extent. Anton Paar, a leading manufacturer of vibrating tube densimeters, distributed the DMA 602 HTP model rated for temperatures from (73.15 to 423.15) K. However, its distribution has been discontinued, and it is likely that the model was never used at temperatures below 243.15 K [7]. The first applications of a VTD at temperatures as low as 240 K have been presented by Kayukawa *et al.* [8–10] and Kano *et al.* [11], where density measurements were performed using a modified DMA 512 of Anton Paar. More recently, Jiao *et al.* [12] published an extended calibration of a VTD for temperatures from (203 to 423) K. The same system was later used for measurements in the temperature range from (203 to 293) K by Tenardi *et al.* [13]. However, the utilized DMA HPM densimeter of Anton Paar is only rated for temperatures of (263.15 to 473.15) K. For comprehensive density measurements within this specified temperature range, typical estimates for the expanded uncertainty in density (*k* = 2) are between (1.0 and 2.0) kg·m^−3^ [14–16] or (0.1 and 0.2)% [17, 18]. The work of Jiao *et al.* [12] and Tenardi *et al.* [13] at temperatures well below this specification yielded uncertainties in a comparable magnitude of (0.31 to 0.42)% and 1.0 kg·m^−3^, respectively. Thus, reliable functionality of the DMA HPM has been demonstrated for temperatures as low as 203 K.

In general, the DMA HPM, which is widely used in the literature [12–38], appears to have the potential to be used for reliable density measurements at temperatures even below 203 K. This would allow for low-temperature density measurements that are significantly more accessible and easier to operate than measurements with gravimetric precision densimeters. Low-temperature vibrating tube densimeters could be used in laboratory applications and in the field, where high-precision densimeters cannot be readily utilized. Potential cryogenic applications [39] would be, for example, investigations of new or improperly studied refrigerants [11–13], process monitoring and quality assurance of cryogenic product chains, or density measurements along the liquefied natural gas value chain [40]. Therefore, in this work, a targeted low-temperature study has been performed to specifically evaluate the functionality and accuracy of the DMA HPM temperatures as low as 100 K.

## Experimental Section

### Apparatus Description

**Fig. 1:**
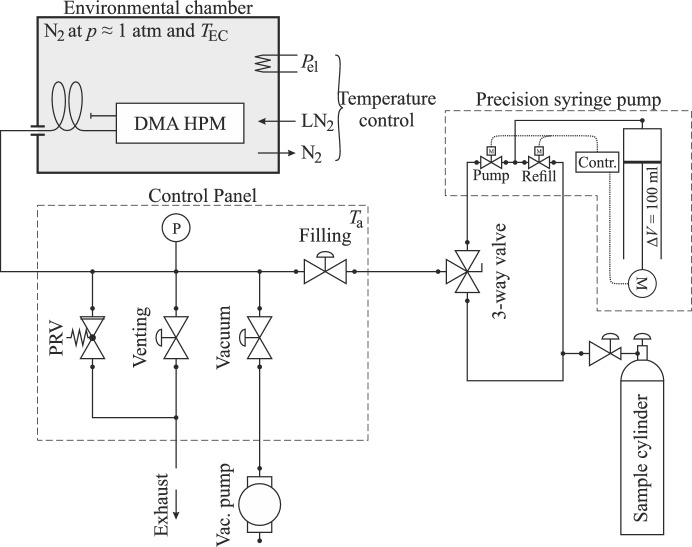
Schematic diagram of the experimental setup, including the environmental chamber (top left), control panel (bottom left), and the sample inlet circuit composed of the sample cylinder, precision syringe pump, and 3-way valve (right)

The new measurement system used for the presented low-temperature study enables investigations at temperatures from (88 to 473) K and from vacuum to pressures of up to 11 MPa. A schematic diagram of the experimental setup commissioned is given in Fig. 1.

The vibrating tube densimeter used (Anton Paar, Austria, type: DMA HPM) is rated for temperatures from (263 to 473) K at pressures up to 140 MPa [41]. The densimeter unit features thermostat channels and corresponding hose couplings for temperature control with an external thermostat. For this study, however, the densimeter is housed in an environmental chamber (inTEST Thermal Solutions, USA, type: M58) without any modification to the VTD unit. The environmental chamber is placed on a structural frame of aluminum profiles that also holds the measurement electronics. By injecting liquid nitrogen (LN_2_) in combination with electrical heating elements, the chamber can provide a temperature-controlled dry nitrogen atmosphere with temperatures from *T*_EC_ = (88 to 473) K, achieving a stability mostly within 0.1 K. Since the environmental chamber is equipped with radial fans to circulate the atmosphere, the densimeter is placed on damping pads to decouple it from any external vibrations and to ensure contact with the atmosphere in the environmental chamber on all sides. The oscillation period of the VTD is recorded and displayed by an evaluation unit (Anton Paar, Austria, type: mPDS 5), which, together with the interface module, is placed outside the environmental chamber (not shown in Fig. 1). The metal-braided cable connecting the VTD and the interface module, as well as the sample inlet tube, are guided through a door notch of the environmental chamber. The inlet tube is connected to a control panel that is mounted to the structural frame of the setup. Because the fluids investigated in this study are gaseous under ambient conditions, they can be removed from the system by evacuation. Therefore, no flushing of the VTD is required for a change of the fluid under survey, and the outlet of the VTD could be blind plugged. The control panel incorporates a pressure transmitter, a proportional pressure relief valve (PRV) as overpressure protection, and three valves to fill, vent, and evacuate the system. The filling valve is connected to the sample inlet circuit that comprises the sample cylinder and an electric syringe pump (Teledyne Isco, USA, type: 100DM). Employing a 3-way valve, the control panel can be connected to the syringe pump or the sample cylinder, depending on the required pressures and the fluid to be filled in. When the pressure of the sample cylinder is below the target-filling pressure, the syringe pump can be used to increase the pressure in the measuring system. A rotary vane pump (Pfeiffer Vacuum, Germany, type: Duo 6) equipped with a zeolite sorption trap is used to evacuate the apparatus.

The pressure transmitter (Keller, Switzerland, type: PAA-33X) with a working range of (0 to 40) MPa has been calibrated with a piston gauge (DH Budenberg, Germany, type: DH 5201) from *p* = (0.2 to 10.0) MPa. The (0 to 10) V signal of the transmitter is measured with a digital multimeter (Tektronix, USA, type: DMM 6500). The expanded uncertainty (*k* = 2) of the pressure measurement was estimated to be within 65 hPa.

The DMA HPM features an internal 1 kΩ platinum resistance thermometer of which temperature is read by the evaluation unit with a stated uncertainty of < 0.1 K. To obtain lower uncertainties, an external 100 Ω platinum resistance capsule thermometer (Minco, USA, type: S1059PA) is installed in a respective 4 mm bore of the VTD block. The capsule thermometer with an outer diameter of 3.2 mm and a length of 9.7 mm was mounted on a custom-made bracket that allows secure installation of the thermometer in the 130 mm deep bore of the VTD. Using a copper sleeve and cryogenic thermal grease, a seating with good heat transfer could be achieved despite the adaptation of the deviating radii of the bore and capsule thermometer. The resistance of the thermometer is measured with a precision bridge (Isotech, USA, type: milliK). The thermometer has been calibrated with a reference thermometry chain in the temperature range from *T* = (103 to 303) K. The expanded uncertainty (*k* = 2) of the entire temperature measurement chain was estimated to be 35 mK.

### Measuring Principle

The measuring principle of vibrating tube densimeters is based on the fundamental bending mode of a hollow tube bent into a ‘U’ shape. This principle for measuring densities was developed by Kratky *et al.* [42] in 1969 and has been developed further since. The U tube, clamped in a counterweight to isolate it from external vibrations, is being excited using a wire-coil electromagnet, and the resulting resonance oscillation period is monitored with a second electromagnet and a frequency counter. When the tube is filled with a gas or liquid, the oscillation period increases, and the higher the density of the fluid ${\rho }_{{\text{fluid}}}$, the longer the oscillation period $\tau$ becomes. The classical working equation for vibrating tube densimeters is derived from the model of an undamped spring–mass system [42] and yields the relation

1 $${\rho }_{{\text{fluid}}}\left(p,T\right)=A\left(p,T\right)\cdot {\tau }^{2}-B\left(p,T\right).$$

The parameters $A\left(p,T\right)$ and $B\left(p,T\right)$ vary with temperature $T$ and pressure $p$ and can be calculated with various available calibration models (see Sect. 3). In general, their dependencies on temperature and pressure are determined through calibration measurements with reference fluids and, depending on the applied model, by measurements under vacuum. Detailed information on the working principle and developments of vibrating tube densimeters is given by González-Salgado *et al.* [5] and Majer and Pádua [6].

### Experimental Procedures

As stated above, calibrating a vibrating tube densimeter relies on measurements with reference fluids of well-known density. The purity of those reference fluids is essential to accurately determine the densimeter's characteristics. Measurements follow an established preparation procedure to avoid impurities inside the system, e.g., residual samples from previous fillings. In this regard, the apparatus is alternately evacuated and filled with the fluid to be measured at least three times to sufficiently remove residual substances. Since the VTD of the presented setup is blind plugged at its outlet port and, thus, is connected to the control panel only with a single tube, blowing out any residual liquids with pressurized gas is not possible. Therefore, it must be ensured that all fluid-filled components are at a sufficiently high temperature so that any liquids can be removed solely with the utilized vacuum pump; e.g., the saturation pressure of propane at *T* = 120 K is approximately 3.0 × 10^−2^ hPa [43], where the rotary vane pump would not be able to evaporate remaining liquid.

After repeated evacuating and filling cycles, the system is filled to the target pressure, which was chosen to be 10 MPa for the present work. If the respective target pressure cannot be reached by the pressure provided by the sample cylinder, the precision syringe pump can be utilized to raise the system pressure beyond the cylinder filling pressure. This is usually necessary for two-phase fluids that are present at their respective saturation pressure. In this case, the sample cylinder is heated to (303 to 313) K using a heating jacket. Consequently, the pressure in the cylinder increases to the corresponding saturation pressure. Afterward, the apparatus is filled to this pressure directly from the cylinder by setting the 3-way valve to the corresponding position (see Fig. 1). When the environmental chamber is kept at ambient temperature or below, the gas introduced condenses inside the entire system, including the control panel, which is kept at ambient temperature. Subsequently, the syringe pump was also filled directly from the cylinder. Then, the syringe pump is connected to the control panel via the 3-way valve. Since the entire apparatus is filled with liquid at this point, the system pressure can be increased to 10 MPa with just a small displacement of the syringe pump. When this exact sequence is applied, the sample in the VTD comes directly from the sample cylinder without passing through the syringe pump. This is advantageous because additional dead volumes and possible contamination with previous measuring fluid can be avoided. However, potential contaminations in the syringe pump may be pumped into the control panel but most likely will not enter the VTD due to the low compressibility of liquids and the comparatively long sample inlet tube that is routed in two loops within the environmental chamber (see Fig. 1). For example, the pressure of liquid propane can be increased from 1.3 MPa to 10.0 MPa with a displacement of the syringe pump of less than 15 ml, which is less than the fluid-filled volumes of the filling line and the control panel.

After filling the apparatus to the pressure of ${p}_{{\text{max}}}$ ≈ 10 MPa, the environmental chamber is set to the target temperature with a cooling rate of maximal 3 K·min^−1^. When the drifts in temperature, pressure, and oscillation period within ten minutes are less than 10 mK, 50 hPa, and 0.01 µs, respectively, the system is considered sufficiently equilibrated, and measurement data at the first state point is recorded. Measurements of further state points are subsequently performed along the corresponding isotherm at pressures of approximately (8, 6, 4, and 2) MPa. Pressure steps are accomplished by venting sample from the system or using the precision syringe pump when the system pressure is above the saturation pressure of the respective temperature. For every state point, temperature, pressure, and oscillation period are recorded and averaged over a measuring time of 10 min. The system is evacuated at the end of each isotherm, and a measurement with an evacuated U tube is carried out (see Sect. 4.1) over a measuring time of 20 min. If the liquid studied cannot be removed solely by the rotary vane vacuum pump, the system is heated to a certain extent until a complete boil-out can be observed. Then, the VTD is cooled back to the temperature of the investigated isotherm and the vacuum measurement is performed. Note: even when the saturation pressure of the fluid is above the final pressure of the vacuum pump, due to the metastable behavior of liquids [44–46], it may be necessary to further heat the system until the boiling begins.

## Calibration Models

The temperature- and pressure-dependent parameters $A$ and $B$ of the classical working equation for vibrating tube densimeters, as given in Eq. 1, can be determined with various calibration models. Anton Paar, the manufacturer of the VTD used, recommends polynomials to calculate these parameters [41]:

2 $$A\left(p,T\right)={A}_{1}+{A}_{2}T+{A}_{3}p+{A}_{4}{T}^{2}+{A}_{5}{p}^{2}$$

and

3 $$B\left(p,T\right)={B}_{1}+{B}_{2}T+{B}_{3}p+{B}_{4}{T}^{2}+{B}_{5}{p}^{2}+{B}_{6}{p}^{4}.$$

This model with 11 adjustable parameters represents the apparatus parameters without physical background. Through calibration measurements with reference fluids, parameters ${A}_{i}$ and ${B}_{i}$ can be adjusted for a best fit of densities calculated according to Eqs. 1–3 to densities calculated with state-of-the-art equations of state. A similar polynomial approach was presented by Outcalt and McLinden [47]. Their model, derived from the work of Ihmels and Gmehling [48], involves 16 adjustable parameters to determine $A$ and $B$ as follows:

4 $$A\left(p,T\right)=\frac{{A}_{1}+{A}_{2}T+{A}_{3}{T}^{2}+{A}_{4}{T}^{3}+{A}_{5}p+{A}_{6}{p}^{2}+{A}_{7}Tp}{{\left({c}_{0}+{c}_{1}T+{c}_{2}{T}^{2}\right)}^{2}}$$

and

5 $$B\left(p,T\right)={B}_{1}+{B}_{2}{T}^{2}+{B}_{3}{T}^{3}+{B}_{4}p+{B}_{5}{p}^{2}+{B}_{6}Tp.$$

Besides different polynomials for temperature and pressure, the main difference from the method recommended by Anton Paar is the consideration of the period of oscillation of the VTD under vacuum. The parameters ${c}_{i}$ in the denominator of Eq. 4 are fitted to measurements of the evacuated U tube to describe the vacuum resonance ${\tau }_{0}$ as a function of temperature:

6 $${\tau }_{0}\left(T,\rho =0\right)={c}_{0}+{c}_{1}T+{c}_{2}{T}^{2}.$$

Thus, when inserting Eqs. 4 and 6 into Eq. 1, the working equation yields the ratio ${\tau \left(T,p,\rho \right)}^{2}/{\tau }_{0}{\left(T, \rho =0\right)}^{2}$, putting the oscillation of the fluid-filled densimeter directly into relation with the resonance of the evacuated U tube.

A further well-established method to determine parameters $A$ and $B$ is the physically based model by May *et al.* [49, 50]. Its adjustable parameters are related to the geometry, material properties, and fundamentals of a freely vibrating uniform cantilever. Due to its physical basis, the model by May *et al.* [49, 50] requires only seven parameters to relate the measured oscillation period to the fluid density. When relating the model to the working equation as given in Eq. 1, the apparatus-specific parameters are determined as follows:

7 $$A\left(p,T\right)=\frac{{\rho }_{00}}{{{\tau }_{00}}^{2}}\left[\frac{1+{\beta }_{\tau }p}{{\left(1+{\varepsilon }_{\tau 1}t+{\varepsilon }_{\tau 2}t\right)}^{2}\cdot \left(1+{\alpha }_{V}t+{\beta }_{V}p\right)}\right]$$

and

8 $$B\left(p,T\right)=\frac{{\rho }_{00}}{1+{\alpha }_{V}t+{\beta }_{V}p}.$$

Here, ${\tau }_{00}$ is the vacuum oscillation period at a reference temperature ${T}_{0}$, ${\beta }_{\tau },$ and ${\beta }_{V}$ consider the pressure distortion of the U tube, ${\alpha }_{V}$ accounts for changes in the volume of the tube with temperature, and ${\varepsilon }_{\tau 1}$ and ${\varepsilon }_{\tau 2}$ describe the vacuum resonance period as a quadratic function of the temperature. The temperature difference $t=T-{T}_{0}$ relates the measured temperature $T$ from the reference temperature ${T}_{0}$. The dimensionless parameter ${\rho }_{00}$ is a measure of sensitivity; that is, how much the oscillation period depends on the density of the fluid.

In the literature, more models for calibrating a vibrating tube densimeter are available (e.g., [15, 51–54]). However, since this work focuses on the general functionality of the VTD at low temperatures, a comprehensive comparison of different calibration methods was not performed. For the calibration and validation measurements presented in this work, only the models of Outcalt and McLinden [47] and May *et al.* [49, 50] are applied. These models have been used in recent publications [12–15] that used the same DMA HPM model.

## Results

### Vacuum Measurements

As mentioned, the DMA HPM vibrating tube densimeter is only rated for temperatures from (263 to 473) K [41]. However, recent works by Jiao *et al.* [12] and Tenardi *et al.* [13] have already used this VTD model in an environmental chamber at temperatures down to 203 K. Both groups of authors found that results based on evaluations using the model by May *et al.* [49, 50] mostly deviated less than 0.6 % from the reference equations used, demonstrating the functionality of the VTD at these temperatures. To examine whether the applicable temperature range can be extended further, the environmental chamber of the present setup was used to record oscillation periods at successively lower temperatures of the evacuated U tube, in the following referred to as vacuum measurements.

The vacuum oscillation period varies with temperature. Established calibration models (see Sect. 3) represent this dependency with quadratic polynomials [47, 49, 50]. Therefore, a first step was to test whether the results of vacuum measurements at low temperatures comply with this quadratic expression. In the first phase, vacuum measurements were performed at temperatures from (174 to 298 K). Here, the deviations of the measured vacuum periods to a corresponding quadratic fit are between (− 0.040 and 0.046) µs, which is of the typical order of magnitude. Vacuum measurements at lower temperatures were initially impossible because the mPDS 5 evaluation unit switched to an 'inactive' mode once the internal temperature measurement read a value of less than 173.15 K (− 100 °C). In this state, no values for the oscillation period were recorded or displayed and no density measurements could be performed at $T$ < 173.15 K. To solve this issue, following the confidential instructions of the Anton Paar Company, the signal processing of the internal temperature measurement was manipulated so that the evaluation unit now is functional below temperatures of 173.15 K. Consequently, the oscillation period could be monitored, even at temperatures as low as 100 K. Please note that this modification was not done at the VTD unit itself and, therefore, does not affect the measurement of the oscillation period. The VTD and the metal-braided connection cable, operated within the chamber's cryogenic atmosphere, were still used in their stock configurations. Additionally, since an external 100 Ω platin resistance thermometer is used, evaluating the presented results is independent of the internal temperature measurement.

**Fig. 2:**
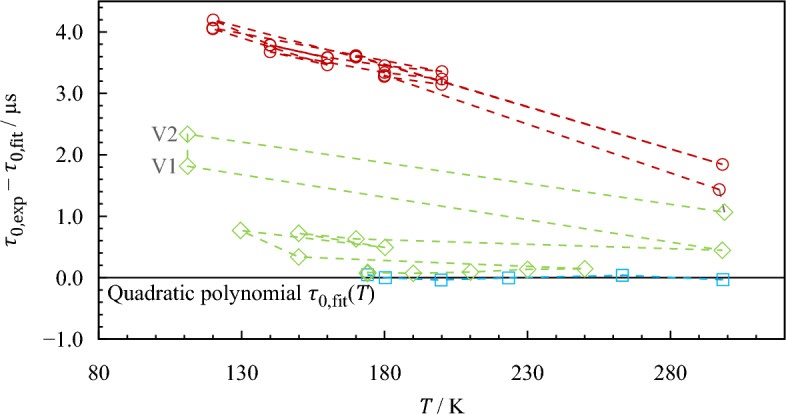
Deviations of selected measured vacuum oscillation periods ${\tau }_{0,{\text{exp}}}$ from values calculated with a quadratic polynomial ${\tau }_{0,{\text{fit}}}\left(T\right)/{{\upmu}}{\text{s}}$ = 2500.859 + $T$/K∙2.346∙10^−1^ + ($T$/K)^2^∙1.794 × 10^−4^ (zero line). , Initial vacuum measurements used to fit the quadratic polynomial; , first vacuum measurements after modifying the internal temperature measurement; , values recorded during the calibration and validation measurements. The connecting lines (- - -) indicate the chronological order of the measurements, starting at the point at 298 K and $\Delta \tau$ ≈ 0.0 µs. V1 and V2 are measurements conducted during stationary operation at constant temperature (see Fig. 3)

Based on this modification, vacuum measurements could be conducted even at temperatures down to 100 K, which was the lowest temperature applied within the present study. Even at this temperature, the DMA HPM continued to operate with plausible responses. However, when the newly recorded values are compared with the previously determined quadratic polynomial, it is noticeable that the vacuum characteristic of the VTD changes, i.e., the temperature-dependent vacuum oscillation period increases over time. This behavior occurred during all subsequent performance tests at low temperatures, even for the calibration performed later (see Sect. 4.2). To illustrate this phenomenon, Fig. 2 shows a selection of vacuum measurements and their deviations from a quadratic polynomial ${\tau }_{0,{\text{fit}}}$($T$), determined with the initial vacuum measurements carried out before the modification ( in Fig. 2). The diamond symbols () represent the first set of measurements with the modified evaluation unit. Subsequently, the system was exposed to multiple thermal cycles within a temperature range from (100 to 300) K to deliberately trigger the changing vacuum characteristics (not shown in Fig. 2). The vacuum periods recorded during the actual calibration and validation measurements are marked with circles ().

**Fig. 3:**
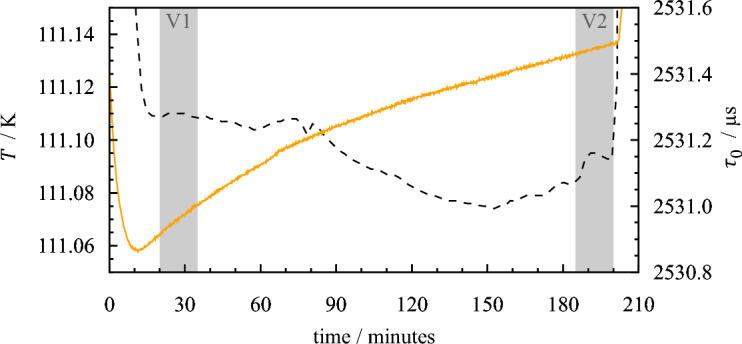
Temporal drift of the recorded vacuum oscillation period ${\tau }_{0}$ at $T$ ≈ 111 K. ‑ ‑ ‑, Temperature; , period. , Time periods of the two vacuum measurements V1 and V2 (see also Fig. 2)

Although the extrapolation of the quadratic fit to lower temperatures alone could explain the visible deviations to some extent, it is apparent that the vacuum oscillation period shifted with every low-temperature thermal cycle of the densimeter to temperatures below approximately 180 K. This effect can be observed especially when comparing the values measured at approximately 300 K. Here, the overall change from the initially determined quadratic fit amounts to more than 1.8 µs. Using the parameters derived from the calibration described later in Sect. 4.2, this shift of the vacuum oscillation period would cause an intolerable change of determined densities of approximately 24 kg·m^−3^. Figure 3 shows an illustrative time frame in which a significant temporal drift of the oscillation period occurred. During 3 h at an almost constant temperature of about 111 K, two 15-min averages were determined (marked in gray). It can be seen that the values differ by more than 0.5 µs, even though the temperature difference between the two measurements is less than 20 mK. These two measurements (V1 and V2) are indicated in Fig. 2.

For the subsequent measurements, such a pronounced drift was not observed under stable conditions again. The shift of the vacuum oscillation period with thermal cycles diminished but never came to a complete stop, even after deliberate thermal cycling. In total, the VTD has been subjected to more than 15 cooling cycles to temperatures below 180 K. The ultimate cause for the shift of the vacuum oscillation period is unknown. Possible sources might be changes in the material structure of the U tube (comparable to the aging of resistance thermometers) and an according change in the spring constant or the build-up or release of tension at the clamping of the counterweight. Similar changes in vacuum characteristics were reported by Laznickova and Huemer [55]. They observed a decrease of ${\tau }_{0}$ with time and thermal load for two types of vibrating tube densimeters; one consisting of a glass tube and one with a tube made of metal (1.4571). They propose a recalibration of the vacuum oscillation period for each measurement. This approach is also followed by several studies in the literature in which a DMA 512 of Anton Paar was used. Kayukawa *et al.* [9, 10] performed measurements of the evacuated U tube at the end of each investigated isotherm. Bouchot and Richon [51] consider the temporal shift of ${\tau }_{0}\left(T\right)$ by recalibrating the vacuum oscillation at a reference temperature, and Kano *et al.* [11] account for the aging of their densimeter using the most recent vacuum values.

Considering the DMA HPM densimeter, Jiao *et al.* [12] tested the long-term stability of ${\tau }_{0}$ during the duration of their 6-month measurement program. Here, it was found that the vacuum oscillation period at $T$ = 204 K remained stable within 0.013 µs. For the present study, this stability was not achieved. It may be that the observable shift will come to a standstill when the VTD unit is exposed to more thermal cycles. Depending on the cause of this instability, it may also happen that the VTD stops operating reliably at some point. Further tests or disassembling of the VTD unit may be required, to ultimately identify the cause and to check if the phenomenon can be prevented, that is, by 'pre-aging' the unit with imposed thermal cycles. However, with the currently persistent shifts of ${\tau }_{0}$, the challenge is that this inconsistency limits the potential accuracy of density measurements. To easily adjust for these shifts, Holcomb and Outcalt [52] formulated their calibration equation relative to a vacuum reference period. This relation of ${\tau \left(T,p,\rho \right)}^{2}/{\tau }_{0}{\left(T, \rho =0\right)}^{2}$ is also included in the models of May *et al.* [49, 50] and Outcalt and McLinden [47] (see Sect. 3). However, they propose a calculation of ${\tau }_{0}$ with quadratic polynomials (e.g., Eq. 6) that will not be representable for subsequent measurements, when the VTD is subject to a persistent shift of the vacuum oscillation period.

This can be compensated for by performing a vacuum measurement directly after the density measurement. That measured value then constitutes the representative vacuum characteristics of the VTD at the time of the density measurement. Since the fluid measurements are carried out along isotherms (see Sect. 2.3), a vacuum measurement can be performed at the end of each isotherm [9, 10]. To exclude the possibility that the vacuum period changes during the measurements for a single isotherm, a vacuum measurement was made each before and after measurements on methane along an isotherm at $T$ = 120 K. Both recorded mean values comply within 0.012 µs. Therefore, by measuring the vacuum period after each isotherm, every density measurement can be related to a valid value of ${\tau }_{0}$. As the temperature along an isotherm is not exactly constant (deviations of less than 85 mK), the recorded vacuum oscillation period is corrected by the temperature difference to the respective measured state point. As an approximation for the sensitivity $\partial {\tau }_{0}/\partial T$, the temperature derivative of the quadratic polynomials determined by the calibration models is used. With this experimental consideration of the vacuum oscillation period, the determination of the calibration parameter (*p*, *T*) according to Eqs. 4 or 7 is modified so that the quadratic polynomials for the description of ${\tau }_{0}\left(T\right)$ are substituted with the corrected experimental value of the vacuum oscillation period. These variations of the calibration models are referred to as modified models in the following. The calibration parameter *A*(*p*, *T*) then can expressed as follows:

9 $$A\left(p,T\right)=\frac{{A}_{1}+{A}_{2}T+{A}_{3}{T}^{2}+{A}_{4}{T}^{3}+{A}_{5}p+{A}_{6}{p}^{2}+{A}_{7}Tp}{{\left({\tau }_{0,{\text{exp}}}+\left(T-{T}_{0,{\text{exp}}}\right)\frac{{\partial \tau }_{0}}{\partial T} \right)}^{2}}$$

for the modified Outcalt and McLinden [47] model and as follows:

10 $$A\left(p,T\right)={\rho }_{00}\left[\frac{1+{\beta }_{\tau }p}{{\left({\tau }_{0,{\text{exp}}}+\left(T-{T}_{0,{\text{exp}}}\right)\frac{{\partial \tau }_{0}}{\partial T}\right)}^{2}\cdot \left(1+{\alpha }_{V}t+{\beta }_{V}p\right)}\right]$$

for the modified May *et al.* [49, 50] model. Here, ${\tau }_{0,{\text{exp}}}$ is the measured value of the vacuum oscillation period measured at the temperature ${T}_{0,{\text{exp}}}$. These modifications replace three adjusting parameters of each of the models that originally describe the vacuum characteristic as quadratic polynomials (${c}_{0}$, ${c}_{1}$, and ${c}_{2}$ for the model by Outcalt and McLinden [47] and ${\tau }_{00}$, ${\varepsilon }_{1}$, and ${\varepsilon }_{2}$ for the model by May *et al.* [49, 50]). Therefore, the modified Outcalt and McLinden [47] model now uses 13 adjustable parameters while only 4 parameters are utilized when applying the modified May *et al.* [49, 50] model.

Clearly, these measures of performing a vacuum measurement after each isotherm lead to additional experimental efforts. But this way, the density measurements do not rely on quadratic fits, which were found to be unsuitable for the present low-temperature study. The results of the vacuum measurements carried out during the calibration and validation measurements (see Sect. 4.2) and the values calculated with a quadratic polynomial are listed in Table 1. As can be seen, the calculated values ${\tau }_{0,{\text{fit}}}$ show absolute deviations of up 0.157 µs from the experimentally determined values ${\tau }_{0,{\text{exp}}}$. Here, a higher value for the vacuum period translates into a decrease in the calculated density, and conversely.

**Table 1:** Results of the vacuum measurements recorded after the isotherms of each reference fluid used for the calibration and validation measurements (see Sect. 4.2), where ${T}_{0,{\text{exp}}}$ is the temperature, ${\tau }_{0,{\text{exp}}}$ is the measured vacuum oscillation period, and ${\tau }_{0,{\text{fit}}}$ is the oscillation period calculated with the quadratic fit

${T}_{0,{\text{exp}}}$(K)	${\tau }_{0,{\text{exp}}}$(µs)	${\tau }_{0,{\text{fit}}}\left({T}_{0,{\text{exp}}}\right)$ ^a^ (µs)	${\tau }_{0,{\text{fit}}}-{\tau }_{0,{\text{exp}}}$(µs)
Methane			
200.06	2558.340	2558.289	− 0.051
180.04	2552.374	2552.300	− 0.074
160.05	2546.596	2546.536	− 0.060
139.94	2540.996	2540.959	− 0.037
119.91	2535.630	2535.626	− 0.004
Propane			
200.05	2558.216	2558.286	0.070
180.00	2552.251	2552.287	0.036
159.98	2546.457	2546.518	0.061
139.96	2540.892	2540.965	0.073
119.86	2535.627	2535.612	− 0.015
Ethane^b^			
199.98^c^	2558.107	2558.265	0.158
179.91^c^	2552.159	2552.262	0.103
160.05	2546.589	2546.536	− 0.053
139.93	2540.996	2540.956	− 0.040
120.02	2535.804	2535.654	− 0.150
Argon^b^			
169.96	2549.539	2549.367	− 0.172

^a^The calculated values ${\tau }_{0,{\text{fit}}}$ are basically identical for the calibration models as both are least-square fits in the form of a quadratic polynomial. The parameters for the calculation of ${\tau }_{0,{\text{fit}}}$ are given in Table 3

^b^The vacuum oscillation periods recorded after the ethane and argon isotherms were not used for fitting the quadratic polynomials

^c^The measurements on ethane at $T$ = (200 and 180) K were performed before the calibration measurements on methane and propane which is why their corresponding vacuum periods are lower than the values with the quadratic polynomials

### Calibration and Validation

For the determination of the apparatus-specific parameters $A\left(p,T\right)$ and $B\left(p,T\right)$ in Eq. 1, calibration measurements with reference fluids were performed. The choice of reference fluids depends on the substance that will be investigated with the calibrated VTD. The density range of the reference fluids should cover the entire density range of the fluid of interest while preferably being close to the densities to be measured. In many cases, vibrating tube densimeters are calibrated with helium and water over the temperature and pressure range of interest. However, water cannot be used for calibration measurements at temperatures below its melting curve. For their extended calibration in the temperature range of (203 to 423) K, Jiao *et al.* [12] used methane and propane as reference fluids. Tenardi *et al.* [13] calibrated their VTD at temperatures from (203 to 293) K with measurements on nitrogen, water (above 273 K), carbon dioxide, and the refrigerant R32 (difluoromethane). The experimental materials used in this work are specified and described in Table 2.

**Table 2:** Description of the experimental materials used in the present work

Substance	CAS number	Source	Purity (mole fraction)
Methane	74-82-8	Westfalen AG	0.999995^a^
Propane	74-98-6	Matheson Gas	0.999990^b^
Ethane	74-84-0	Matheson Gas	0.999995^c^
Argon	7440-37-1	Air Liquide	0.999999^d^

^a^Impurities (stated by supplier): *x*(O_2_) ≤ 0.5 ppmv, *x*(N_2_) ≤ 4.0 ppmv, *x*(H_2_O) ≤ 2.0 ppmv, *x*(C_n_H_m_) ≤ 0.5 ppmv

^b^Impurities (stated by supplier): *x*(CO_2_) ≤ 1 ppm, *x*(N_2_) ≤ 2 ppm, *x*(O_2_) ≤ 1 ppm, *x*(C_n_H_m_) ≤ 8 ppm,* x*(C_3_H_6_) ≤ 2 ppm,* x*(H_2_O) ≤ 1 ppm

^c^Impurities (stated by supplier): *x*(C_2_H_2_) ≤ 0.5 ppm, *x*(C_4_H_10_) ≤ 0.5 ppm, *x*(CO_2_) ≤ 1.0 ppm, *x*(C_2_H_4_) ≤ 7.0 ppm, *x*(H_2_) ≤ 0.5 ppm, *x*(C_4i_H_10_) ≤ 0.5 ppm, *x*(CH_4_) ≤ 2.0 ppm, *x*(N_2_) ≤ 4.0 ppm, *x*(O_2_) ≤ 2.0 ppm, *x*(C_3_H_8_) ≤ 10.0 ppm, *x*(C_3_H_6_) ≤ 17.0 ppm, *x*(H_2_O) ≤ 4.0 ppm

^d^Impurities (stated by supplier): *x*(H_2_O) ≤ 0.5 ppm,* x*(O_2_) ≤ 0.1 ppm,* x*(CO) ≤ 0.1 ppm,* x*(CO_2_) ≤ 0.1 ppm,* x*(C_n_H_m_) ≤ 0.1 ppm,* x*(H_2_) ≤ 0.1 ppm,

Since the study presented in this paper seeks to assess the performance of the DMA HPM at temperatures as low as 100 K, a sophisticated validation of the calibrated VTD was required. It was found that the DMA HPM keeps operating even at 100 K, with some constraints (see Sect. 4.1). A temperature range from (120 to 200) K has been chosen for calibration due to the assumption that operating the VTD at 100 K may result in a further, significant shift of the vacuum oscillation period. To validate the functionality and assess the accuracy of the VTD in this temperature range as meaningful as possible, the uncertainties of the calculated reference densities need to be as low as possible. Therefore, research-grade methane (purity 5.5, Westfalen AG, Germany) and propane (purity 5.0, Matheson Gas, USA) were used for the calibration measurements. Based on these measurements, the apparatus parameters were determined according to both the models of Outcalt and McLinden [47] and May *et al.* [49, 50]. The reference densities are calculated with their respective reference equations of state. The equation of state for methane by Setzmann and Wagner [56] predicts densities with an uncertainty of 0.03 % for pressures below $p$ = 12 MPa and temperatures below $T$ = 350 K. For the propane equation of state, Lemmon *et al.* [43] report an uncertainty of 0.01 % for liquid densities below $T$ = 350 K.

**Fig. 4:**
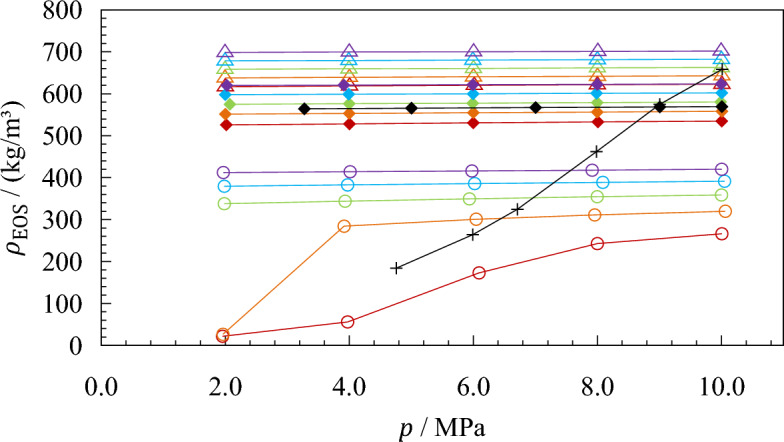
($p$, $\rho$, $T$) state points of the densities recorded for calibration and validation measurements. Densities ${\rho }_{{\text{EOS}}}$ at the ordinate are calculated with the respective reference equations of state [43, 56–58]. ○, Methane; △, propane; ◆, ethane; +, argon. Red, $T$ = 200 K; orange, $T$ = 180 K; black, $T$ = 170 K; green, $T$ = 160 K; blue, $T$ = 140 K; purple, $T$ = 120 K (Color figure online)

The measuring schedule for the calibration and validation measurements is plotted in Fig. 4. For the calibration, methane and propane were investigated along five isotherms of $T$ = (120, 140, 160, 180, and 200) K at pressures of approximately $p$ = (10, 8, 6, 4, and 2) MPa. The 50 resulting state points cover a liquid density range from (284 to 702) kg·m^−3^. In the case of methane, the 200 K isotherm is supercritical and the state point of $p$ = 2 MPa at *T* = 180 K is in the gas phase. These points cover densities from (22 to 267) kg·m^−3^. To validate the calibration of the densimeter, validation measurements on ethane (purity 5.5, Matheson Gas, USA) were performed across the entire temperature and pressure range of the calibration, covering densities between (526 and 624) kg·m^−3^. The experimentally determined densities are compared with the reference equation of state by Bücker and Wagner [57], which has a stated uncertainty of 0.02 % to 0.04 % for densities below $T$ = 520 K and $p$ = 30 MPa. In addition, densities of ethane and argon (purity 6.0, Air Liquide, France) were measured along a further isotherm at $T$ = 170 K at deviating pressures to test the interpolation performance of the calibration models concerning temperature and pressure. Using argon, which is gaseous at this temperature, almost the entire calibrated density range can be validated with a single isotherm. The argon densities are within (183 to 659) kg·m^−3^ and are compared with the equation of state by Tegeler *et al.* [58]. The uncertainty of densities calculated from this equation of state is 0.02 % for the investigated temperatures and pressures.

The parameters of both calibration models were determined with a non-linear least-squares method, considering the absolute deviations of the densities determined with the recorded ($p$, $T$, $\tau$) data from the densities calculated with the respective reference equations of state. Here, only the methane and propane measurements were used for fitting; i.e., the apparatus-specific parameters were not adjusted to the ethane or argon data.

As described in Sect. 4.1, a shift of the vacuum oscillation period was observed when the VTD has undergone low-temperature thermal cycles to temperatures below circa 180 K. This implies that the quadratic expression for the vacuum period used in the calibration models may not be representative of the measurements carried out. Therefore, two sets of calibration parameters, listed in Table 3, were determined for each model. One set that applies the models with the proposed quadratic fits of the oscillation period ${\tau }_{0,{\text{fit}}}$, and one parameter set for the modified models, which has been determined with experimental values ${\tau }_{0,{\text{exp}}}$, measured directly after the respective isotherm. The results of the validation measurements on ethane and argon when applying the modified calibration models are listed in Table 4. The densities for the calibration measurements on methane and propane, determined with both modified models using the experimental values ${\tau }_{0,{\text{exp}}}$, are listed in Sect. S1 of the Supplementary Material. The corresponding results when using the original models, where the vacuum oscillation period is calculated with quadratic polynomials, are given in Sect. S2 of the Supplementary Material.

**Table 3:** Calibration parameters for the vibrating tube densimeter used in this work according to the models of May *et al.* [49, 50] and Outcalt and McLinden [47], determined with measurements on methane, propane, and under vacuum

Parameter	Value	Unit	Parameter	Value	Unit
Model by May * et al.*^a^					
For calculated vacuum oscillation periods ${\tau }_{0,{\text{fit}}}={\tau }_{00}\cdot \left(1+{\varepsilon }_{\tau 1}t+{\varepsilon }_{\tau 2}t\right)$ with $t = T-{T}_{0}$					
${\varepsilon }_{\tau 1}$	1.3345 × 10^−4^	K^−1^	${\alpha }_{V}$	1.0556 × 10^−5^	K^−1^
${\varepsilon }_{\tau 2}$	1.0576 × 10^−7^	K^−2^	${\beta }_{V}$	− 3.2470 × 10^−4^	MPa^−1^
${\tau }_{00}$	2582.015	µs	${\beta }_{\tau }$	− 1.6974 × 10^−5^	MPa^−1^
${\rho }_{00}$	1.6840 × 10^4^	kg·m^−3^			
For experimental vacuum oscillation periods ${\tau }_{0,exp}$ (modified model)					
${\rho }_{00}$	1.6807 × 10^4^	kg·m^−3^	${\beta }_{V}$	1.8804 × 10^−4^	MPa^−1^
${\alpha }_{V}$	2.4789 × 10^−5^	K^−1^	${\beta }_{\tau }$	− 3.1693 × 10^−5^	MPa^−1^
Model by Outcalt and McLinden					
For calculated vacuum oscillation periods ${\tau }_{0,{\text{fit}}}={c}_{0}+{c}_{1}T+{c}_{2}{T}^{2}$					
${c}_{0}$	2508.269	µs	${A}_{6}$	− 3.5448 × 10^−1^	kg·m^−3^·MPa^−2^
${c}_{1}$	1.9539 × 10^−1^	µs·K^−1^	${A}_{7}$	4.1894 × 10^−2^	kg·m^−3^·K^−1^·MPa^−1^
${c}_{2}$	2.7308 × 10^−4^	µs·K^−2^	${B}_{1}$	1.6607 × 10^4^	kg·m^−3^
${A}_{1}$	1.6575 × 10^4^	kg·m^−3^	${B}_{2}$	3.2382 × 10^−2^	kg·m^−3^·K^−2^
${A}_{2}$	7.5756 × 10^−1^	kg·m^−3^·K^−1^	${B}_{3}$	− 1.3165 × 10^−4^	kg·m^−3^·K^−3^
${A}_{3}$	2.6654 × 10^−2^	kg·m^−3^·K^−2^	${B}_{4}$	− 9.0613 × 10^−1^	kg·m^−3^·MPa^−1^
${A}_{4}$	− 1.1796 × 10^−4^	kg·m^−3^·K^−3^	${B}_{5}$	− 3.6617 × 10^−1^	kg·m^−3^·MPa^−2^
${A}_{5}$	− 8.8430 × 10^−1^	kg·m^−3^·MPa^−1^	${B}_{6}$	4.3932 × 10^−2^	kg·m^−3^·K^−1^·MPa^−1^
For experimental vacuum oscillation periods ${\tau }_{0,exp}$ (modified model)					
${A}_{1}$	1.6863 × 10^4^	kg·m^−3^	${B}_{1}$	1.6864 × 10^4^	kg·m^−3^
${A}_{2}$	6.6871 × 10^−3^	kg·m^−3^·K^−1^	${B}_{2}$	− 8.5342 × 10^−4^	kg·m^−3^·K^−2^
${A}_{3}$	− 8.1214 × 10^−4^	kg·m^−3^·K^−2^	${B}_{3}$	1.4348 × 10^−7^	kg·m^−3^·K^−3^
${A}_{4}$	− 1.6468 × 10^−7^	kg·m^−3^·K^−3^	${B}_{4}$	− 1.3437	kg·m^−3^·MPa^−1^
${A}_{5}$	− 1.3266	kg·m^−3^·MPa^−1^	${B}_{5}$	2.4663 × 10^−3^	kg·m^−3^·MPa^−2^
${A}_{6}$	5.4695 × 10^−4^	kg·m^−3^·MPa^−2^	${B}_{6}$	2.6766 × 10^−4^	kg·m^−3^·K^−1^·MPa^−1^
${A}_{7}$	− 5.4137 × 10^−5^	kg·m^−3^·K^−1^·MPa^−1^			

^a^Reference temperature $${T}_{0}$$ = 273.15 K

**Table 4:** Results of the validation measurements of ethane and argon, where $T$ is the temperature, $p$ is the pressure, and $\tau$ is the oscillation period. ${\rho }_{{\text{EOS}}}$ is the density calculated with the reference equations of state (Bücker and Wagner [57] for ethane and Tegeler *et al.* [58] for argon), ${\rho }_{{\text{May}}}$ is the experimental density calculated with the modified May *et al.* [49, 50] model (Eqs. 1, 8, and 10 with the parameters for ${\tau }_{0,{\text{exp}}}$ as listed in Table 3) and ${\rho }_{{\text{OM}}}$ is the experimental density calculated with the modified Outcalt and McLinden [47] model (Eqs. 1, 5, and 9 with the parameters for ${\tau }_{0,{\text{exp}}}$ as listed in Table 3)

$T$(K)	$p$/MPa	$\tau$/µs	${\rho }_{{\text{EOS}}}$/(kg·m^−3^)	${\rho }_{{\text{May}}}$ ^a^/(kg·m^−3^)	$\Delta {\rho }_{{\text{May}}}$/(kg·m^−3^)	${\rho }_{{\text{OM}}}$ ^a^/(kg·m^−3^)	${\Delta \rho }_{{\text{OM}}}$/(kg·m^−3^)
Ethane							
199.99	10.003	2598.525	534.81	535.19	0.38	534.87	0.06
199.99	8.008	2598.356	532.76	533.15	0.39	532.85	0.09
199.99	6.000	2598.181	530.62	531.02	0.40	530.74	0.12
199.99	4.001	2597.997	528.41	528.77	0.36	528.50	0.09
199.99	2.016	2597.812	526.13	526.51	0.38	526.23	0.10
179.95	10.006	2594.202	558.14	558.47	0.33	558.30	0.16
179.93	7.996	2594.057	556.52	556.75	0.23	556.66	0.14
179.93	5.999	2593.913	554.85	555.04	0.19	554.97	0.12
179.92	3.999	2593.768	553.13	553.32	0.19	553.25	0.12
179.92	2.000	2593.618	551.36	551.53	0.17	551.44	0.08
160.01	9.990	2590.145	580.43	580.31	− 0.12	580.52	0.09
160.00	7.996	2590.030	579.12	578.99	− 0.13	579.23	0.11
159.99	5.991	2589.913	577.77	577.65	− 0.12	577.89	0.12
159.98	4.000	2589.793	576.40	576.26	− 0.14	576.52	0.12
159.98	2.075	2589.678	575.05	574.94	− 0.11	575.16	0.11
139.93	10.003	2586.055	602.29	602.15	− 0.14	602.19	− 0.10
139.92	7.987	2585.959	601.19	601.09	− 0.10	601.14	− 0.05
139.92	5.995	2585.866	600.10	600.08	− 0.02	600.11	0.01
139.92	3.991	2585.772	598.97	599.05	0.08	599.04	0.07
139.91	2.001	2585.678	597.84	598.01	0.17	597.95	0.11
119.99	9.999	2582.305	623.60	623.18	− 0.42	623.22	− 0.38
119.99	7.998	2582.225	622.70	622.35	− 0.35	622.37	− 0.33
119.98	5.994	2582.144	621.79	621.50	− 0.29	621.50	− 0.29
119.98	3.910	2582.059	620.82	620.60	− 0.22	620.54	− 0.28
119.97	2.026	2581.981	619.95	619.78	− 0.17	619.67	− 0.28
169.96	10.006	2592.311	569.40	569.11	− 0.29	569.31	− 0.09
169.95	9.005	2592.244	568.69	568.32	− 0.37	568.60	− 0.09
169.93	7.005	2592.116	567.22	566.83	− 0.39	567.15	− 0.07
169.93	5.003	2591.987	565.70	565.32	− 0.38	565.66	− 0.04
169.92	3.276	2591.873	564.37	563.99	− 0.38	564.31	− 0.06
Argon							
169.96	10.013	2598.931	658.43	657.88	− 0.55	657.96	− 0.47
169.95	9.005	2592.703	574.78	574.34	− 0.44	574.42	− 0.36
169.94	7.985	2584.300	462.02	461.86	− 0.16	461.93	− 0.09
169.94	6.710	2573.996	324.38	324.37	− 0.01	324.46	0.08
169.94	5.990	2569.453	263.93	263.92	− 0.01	264.02	0.09
169.94	4.756	2563.425	183.86	183.85	− 0.01	183.99	0.13

^a^Due to temperature deviations between the fluid and vacuum measurements, the values of ${\tau }_{0,{\text{exp}}}$ were corrected with ${\tau }_{0}^{\prime}={\tau }_{0,{\text{exp}}}+\left(T-{T}_{0,{\text{exp}}}\right)\cdot \partial {\tau }_{0}/\partial T$. The sensitivity $\partial {\tau }_{0}/\partial T$ is estimated with the temperature derivative of the respective quadratic fit ${\tau }_{0,{\text{fit}}}\left(T\right)$ as given in Table 3

**Fig. 5:**
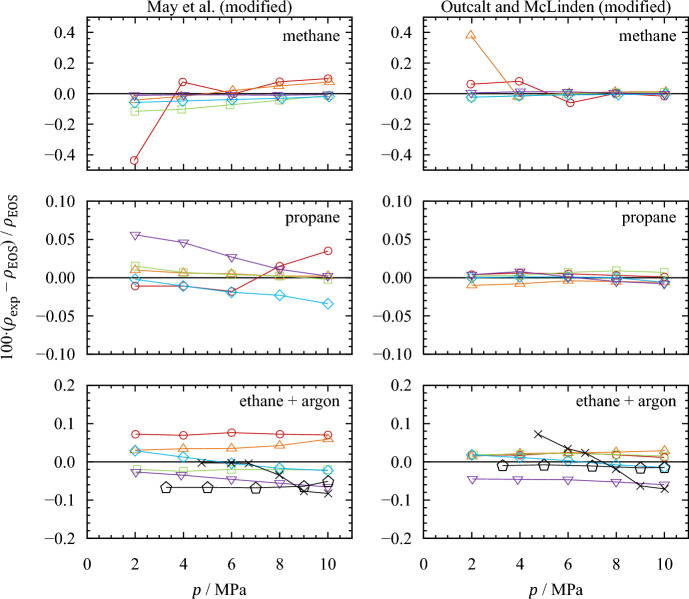
Relative deviations of the experimentally determined densities ${\rho }_{{\text{exp}}}$ (determined with experimental values of ${\tau }_{0,{\text{exp}}}$) from densities ${\rho }_{{\text{EOS}}}$ calculated with the respective reference equations of state (Setzmann and Wagner [56] for methane, Lemmon *et al.* [43] for propane, Bücker and Wagner [57] for ethane, and Tegeler *et al.* [58] for argon). , $T$ = 200 K; , $T$ = 180 K; , $T$ = 160 K; , $T$ = 140 K; , $T$ = 120 K; , $T$ = 170 K (ethane); ✕, $T$ = 170 K (argon)

Figure 5 shows the relative deviations of the experimental densities from densities calculated with the respective reference equation of state when applying the modified calibration models. For methane and propane, the modified May *et al.* [49, 50] model (left) achieves an agreement of measured densities with values calculated with the reference equations of state within ± 0.39 kg·m^−3^. The corresponding relative deviations are (− 0.12 to 0.10) %, except for the state point at $T$ = 200 K and $p$ = 2.0 MPa. For this point, the relative deviation is − 0.44 %. The comprehensive validation measurements with ethane yield relative deviations of (− 0.41 to 0.40) kg·m^−3^ or (− 0.07 to 0.08) % from values calculated with the equation of state by Bücker and Wagner [57]. The measured argon isotherm at *T* = 170 K, which covers almost the entire calibrated density range, shows deviations of (− 0.55 to −0.01) kg·m^−3^ or (− 0.09 to 0.01) % from values calculated with the equation of state by Tegeler *et al.* [58]. The average absolute relative deviation (AARD) of all calibration and validation measurements is 0.039 %.

The calculation of densities according to the modified Outcalt and McLinden [47] model (right in Fig. 5) performs noticeably better. Here, all experimental data points for methane and propane deviate within ± 0.10 kg·m^−3^ from values calculated with the respective equations of state. The deviations for ethane and argon range from (− 0.47 to 0.16) kg·m^−3^. Overall, the relative deviations range from (− 0.07 to 0.08) %, except for the gaseous state point of methane at $T$ = 180 K and $p$ = 2.0 MPa. Here, the absolute deviation is just 0.10 kg·m^−3^, which corresponds to 0.38 % due to the low density of about 26.22 kg·m^−3^. The AARD of all state points in the calibration and validation measurements is 0.022 %.

**Fig. 6:**
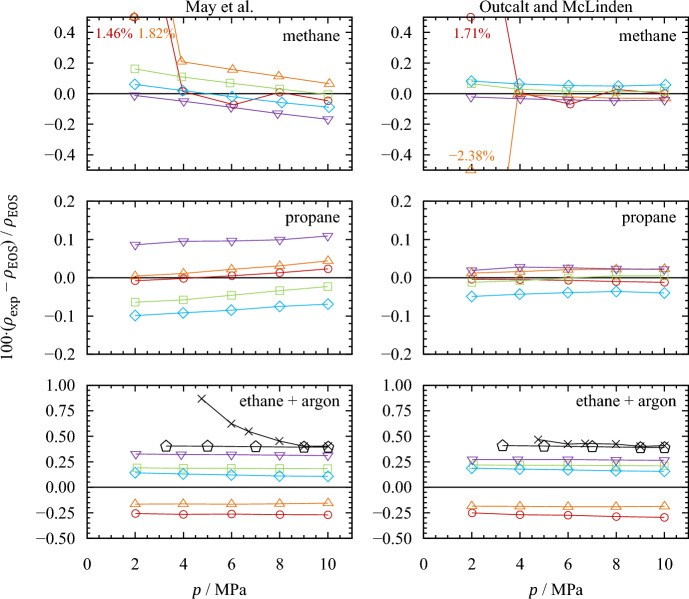
Relative deviations of the experimentally determined densities ${\rho }_{{\text{exp}}}$ [determined with calculated values of ${\tau }_{0,{\text{fit}}}\left(T\right)$, as in Eqs. 4 and 7] from densities ${\rho }_{{\text{EOS}}}$ calculated with the respective reference equations of state (Setzmann and Wagner [56] for methane, Lemmon *et al.* [43] for propane, Bücker and Wagner [57] for ethane, and Tegeler *et al.* [58] for argon). , $T$ = 200 K; , $T$ = 180 K; , $T$ = 160 K; , $T$ = 140 K; , $T$ = 120 K; , $T$ = 170 K (ethane); ✕, $T$ = 170 K (argon)

As mentioned, these results are based on experimental values for the vacuum oscillation period and were, therefore, determined with the modified calibration models (Eqs. 9 and 10). When the temperature-dependent vacuum period is calculated with quadratic polynomials ${\tau }_{0,{\text{fit}}}$, as originally proposed by the authors, both calibration models are unable to determine densities with comparable accuracy. Figure 6 shows the relative deviations from the reference equations of state for this case. Both models are capable of reproducing the reference densities of methane and propane rather well, except for a few state points of methane in the gaseous and supercritical regions. The validation measurements, however, show that the determined calibration parameters are not able to predict the ethane and argon densities with an accuracy similar to that when measured vacuum oscillation periods are used. Here, the overall deviations range from (− 1.44 to 2.65) kg·m^−3^ or (− 0.27 to 1.82) % for the model by May *et al.* [49, 50] and from (− 1.58 to 2.69) kg·m^−3^ or (− 2.38 to 1.71) % for the model by Outcalt and McLinden [47]. The AARDs are 0.20 % and 0.18 %, respectively. For ethane at $T$ = (180 and 200) K, both models result in negative deviations from the equation of state. This is because these isotherms were recorded before the calibration measurements on methane and propane and, thus, at a time with less advanced shift of ${\tau }_{0}$. Consequently, the vacuum oscillation periods calculated with the quadratic polynomials are higher than the values measured at that time (see Table 1), leading to a smaller ratio of ${\tau }^{2}/{\tau }_{0}^{2}$ and likewise lower densities.

The numerical values for this evaluation are listed in Sect. S2 of the Supplementary Material. Depending on the application and the uncertainties aspired, this performance may meet the requirements. However, further long-term tests are advisable to assess the stability of the densimeters' vacuum characteristics. Moreover, a regular recalibration, at least of the vacuum oscillation periods, is recommended.

The comparison of both options for handling the vacuum oscillation shows that accurately determining densities is currently only possible by directly measuring the vacuum oscillation period for each isotherm along with the fluid measurements. This is the case as long as the VTD is subject to a shifting vacuum characteristic. However, a crucial consideration is the long-term stability of the performed calibration; that is, whether the determined characteristics also change so that an accurate determination of fluid densities is no longer possible, even when applying the modified calibration models. To some extent, this can already be assessed with measurements carried out in the present study. Before the calibration and validation measurements, the VTD has been deliberately exposed to numerous thermal cycles (refer to Sect. 4.1). During the last cycles, approximately four months before the actual calibration, first test measurements were carried out on methane at $T$ = (140, 160, and 180) K and on ethane at $T$ = (160 and 140) K. During the months in between, the apparatus has not been operated due to a technical failure of the environmental chamber. Nevertheless, when evaluating the recorded ($p$, $T$, $\tau$) data for these isotherms with the calibration parameters listed in Table 3, the deviations of the determined densities from the respective reference equation of state are between (0.02 and 0.09) % for methane and (− 0.03 and 0.00) % for ethane, when using the modified Outcalt and McLinden [47] model and the respective experimental vacuum oscillation periods. These results imply that no perceptible change in the densimeter characteristics can be observed after 4 months of system downtime, where the VTD was kept at ambient temperature.

### Uncertainty Analysis

The combined expanded uncertainty in density was estimated in accordance with the ‘Guide to the Expression of Uncertainty in Measurement’ [59] (ISO/IEC Guide), known as GUM. The analysis discussed in this section considers the application of the modified calibration models. The uncertainty contributions were estimated individually, assuming that there is no correlation between the sources of uncertainty. The combined uncertainty in density $u\left({\rho }_{{\text{meas}}}\right)$ due to the uncertainties of the measured quantities can be determined by

11 $${u}{\left({\rho}_{{\text{meas}}} \right)}^{2}={\left[u\left(T\right){\left(\frac{\partial \rho }{\partial T}\right)}_{p}\right]}^{2}+{\left[u\left(p\right){\left(\frac{\partial \rho }{\partial p}\right)}_{T}\right]}^{2}+{\left[u\left(\tau \right){\left(\frac{\partial \rho }{\partial \tau }\right)}_{T,p}\right]}^{2}+{\left[u\left({\tau }_{0}\right){\left(\frac{\partial \rho }{\partial {\tau }_{0}}\right)}_{T,p}\right]}^{2},$$

where $u\left(T\right)$, $u\left(p\right)$, and $u\left(\tau \right)$, and $u\left({\tau }_{0}\right)$ are the estimated uncertainties of the measured temperature, pressure, oscillation period, and vacuum oscillation period. For $u\left(\tau \right)$ and $u\left({\tau }_{0}\right)$, the standard deviations of the recorded values are used, which are less than 0.006 µs for all validation measurements. The sensitivity coefficients $\partial \rho /\partial X$ are individually determined for every measurement, using the corresponding reference equation of state and the calibrated VTD parameters, respectively. Further uncertainty contributions result from the equations of state used for the calibration fluids $u\left({\rho }_{{\text{EOS}}}\right)$, the applied calibration model $u\left({\rho }_{{\text{cal}}}\right)$, the reproducibility $u\left({\rho }_{{\text{rep}}}\right)$, and the temperature correction of the measured vacuum oscillation period $u\left({\tau }_{0,{\text{corr}}}\right)$. Including these sources of uncertainty, the total expanded combined uncertainty in density $U\left(\rho \right)$ is determined by

12 $$U\left(\rho \right)=k\cdot \sqrt{u{\left({\rho }_{{\text{meas}}}\right)}^{2}+u{\left({\rho }_{{\text{EOS}}}\right)}^{2}+u{\left({\rho }_{{\text{cal}}}\right)}^{2}+u{\left({\rho }_{{\text{rep}}}\right)}^{2}+{\left[u\left({\tau }_{0,{\text{corr}}}\right){\left(\frac{\partial \rho }{\partial {\tau }_{0}}\right)}_{T,p}\right]}^{2}}.$$

The uncertainty of the reference equations of state $u\left({\rho }_{{\text{EOS}}}\right)$ is chosen to be the maximum absolute uncertainty of the investigated state points, which is 0.126 kg·m^−3^ (*k* = 1) for methane at 120 K and 10.0 MPa. The uncertainty of the calibration model $u\left({\rho }_{{\text{cal}}}\right)$ could be defined as a corrected sample standard deviation using the differences between the experimental densities of the calibration measurements and the calculated reference densities. However, the experimental results do not correspond to a normal distribution. Hence, as a conservative estimate, the uncertainty of the calibration is specified as twice the value of the maximum deviation between the experimentally determined densities and the reference densities. This contribution depends on the applied calibration model. Using the measured values ${\tau }_{0,{\text{exp}}}$, the modified May *et al.* [49, 50] model shows a mean absolute deviation of 0.038 kg·m^−3^ and a maximum deviation of 0.392 kg·m^−3^ for the measurements on methane and propane. Consequently, the uncertainty contribution of the calibration (*k* = 1), in this case, is $u\left({\rho }_{{\text{cal}}}\right)$ = 0.784 kg·m^−3^. The modified Outcalt and McLinden [47] model yields $u\left({\rho }_{{\text{cal}}}\right)$ = 0.208 kg·m^−3^ (*k* = 1), whereas the mean absolute deviation of the experimental densities from the respective equation of state is 0.012 kg·m^−3^. For most investigated ($p$, $\rho$, $T$) state points this uncertainty source contributes more than 60 % to the combined uncertainty. Only at low densities in the supercritical and gas regions, other uncertainty sources such as temperature and pressure measurement can contribute to a similar degree. Accordingly, the higher value of $u\left({\rho }_{{\text{cal}}}\right)$ for the modified May *et al.* [49, 50] model results in significantly higher expanded combined uncertainties of the measured densities.

The uncertainty caused by the reproducibility in measurement (*k* = 1.73) was estimated to be equivalent to 0.010 μs. Lastly, the uncertainty of the correction of the experimentally determined vacuum oscillation period $u({\tau }_{0,{\text{corr}}})$ was estimated to be 20 % (*k* = 1.73), considering that vacuum measurements before and after a methane isotherm at 120 K showed an excellent agreement within 0.012 μs. The maximum temperature difference of all fluid measurements to their corresponding vacuum measurements was less than 85 mK, which translates to a maximal correction of ${\tau }_{0}$ of 0.025 μs.

Based on the described analysis, the uncertainty was individually determined for every recorded state point. When applying the modified May *et al.* [49, 50] model, the expanded combined uncertainties in the density range from (0.257 to 0.304) % with an absolute uncertainty of more or less constant 1.60 kg·m^−3^ for the validation measurements on ethane. In the case of argon, the uncertainties range from (1.64 to 1.89) kg·m^−3^ or (0.272 to 0.894) %, respectively. For the modified Outcalt and McLinden [47] model, due to the smaller value of $u\left({\rho }_{{\text{cal}}}\right)$, the uncertainties of the determined ethane densities are about 0.53 kg·m^−3^ or (0.084 to 0.100) %, respectively. The uncertainties of the argon densities are likewise smaller with (0.64 to 1.13) kg·m^−3^ or (0.145 to 0.350) %. For both models, all determined ethane and argon densities agree with the corresponding equation of state well within the experimental uncertainty. An exemplary uncertainty budget for a selected density measurement of ethane is given in Table 5. A list of all uncertainties for the individual state points of the validation measurements is given in Sect. S3 of the Supplementary Material.

**Table 5:** Estimated uncertainty budget for the density measurements. As an example, the uncertainty was calculated for ethane at $T$ = 169.93 K, $p$ = 5.003 MPa, and ${\rho }_{{\text{OM}}}$ = 565.66 kg·m^–3^ (see Table 4)

Source	Expanded uncertainty	*k*	Standard uncertainty in density	Contribution to $U\left(\rho \right)$
Temperature	35 mK	2	0.020 kg·m^−3^	0.58 %
Pressure	65 hPa	2	0.002 kg·m^−3^	0.01 %
Period	0.002 µs	1	0.027 kg·m^−3^	1.04 %
Vacuum Period	0.003 µs	1	0.041 kg·m^−3^	2.42 %
${\tau }_{0}$ correction^b^	0.005 µs	$\sqrt{3}$	0.036 kg·m^−3^	1.88 %
Reproducibility	0.010 µs	$\sqrt{3}$	0.078 kg·m^−3^	8.71 %
Reference EOS	0.126 kg·m^−3^	1	0.126 kg·m^−3^	22.94 %
Calibration^a^	0.208 kg·m^−3^	1	0.208 kg·m^−3^	62.42 %
Combined expanded relative uncertainty $U\left(\rho \right)$ (*k* = 2)				0.526 kg·m^–3^, or 0.093 %

^a^Contribution determined with Eq. (5) for the calibration according to the modified Outcalt and McLinden [47] model

^b^For this specific density measurement, the temperature difference between the fluid and vacuum measurements is ($T$ − ${T}_{0,{\text{exp}}}$) = − 79 mK and the correspondingly applied correction of ${\tau }_{0}$ is − 0.023 µs

## Comparison to a Primary Single-Sinker Densimeter

The results of the calibration and validation measurements show that the vibrating tube densimeter utilized is capable of reliably determining the densities of pure fluids when vacuum measurements are performed after every investigated isotherm. The estimated uncertainty of less than 1.0 kg·m^−3^ for liquids when using the modified Outcalt and McLinden [47] model constitutes an accuracy sufficient for many technical applications. But VTDs also offer significant advantages in the field of fluid science compared to other density measurement principles, even though the uncertainty is considerably higher than for state-of-the-art densimeters. Detailed overviews of density measurement principles are provided by Goodwin *et al.* [1] and Wilhelm and Letcher [2]. In this section, however, the commissioned cryogenic VTD setup is compared directly with a single-sinker gravimetric densimeter at the Thermodynamics Lab of Ruhr University Bochum, Germany.

This single-sinker cryogenic densimeter is based on the Archimedean principle using a magnetic suspension coupling in conjunction with an analytical balance [60, 61]. This precision apparatus employs a well-known, metrologically traceable primary measuring principle. General descriptions of this kind of densimeter are given by Wagner and Kleinrahm [3] and McLinden [4]. The single-sinker densimeter referred to was deliberately developed for accurate density measurements of cryogenic liquefied gas mixtures, i.e., liquefied natural gas-like mixtures. In recent years, it has been used in various measurement campaigns and has provided reference densities for synthetic natural gases, natural gas-like binary mixtures, and other cryogenic mixtures [60–67]. The published data were successfully used to validate and develop state-of-the-art fundamental equations of state and empirical correlations for natural gases and similar mixtures [68, 69]. Covering a temperature range of (100 to 300) K at pressures up to 12 MPa, it is in this regard very comparable to the experimental system used in the present work. Accordingly, both systems can theoretically be used for similar density measurements. Hence, the strengths and drawbacks of the commissioned VTD setup compared to the single-sinker densimeter for different criteria are briefly discussed.

### Uncertainty in Measurement

One of the most decisive specifications in measurements is the associated uncertainty of the reported data. For most applications, the uncertainties required dictate the choice of the measurement principle to be used. In this regard, it is apparent that VTD systems will not be able to achieve the level of precision of sophisticated gravimetric magnetic suspension densimeters. Typical expanded combined uncertainties (*k* = 2) of the single-sinker cryogenic densimeter are less than 0.02 %, for liquid densities of pure fluids or high-quality gas mixtures [61, 62]. Regarding density measurements in the gaseous or supercritical phase, or when using mixtures with higher uncertainties in composition, the uncertainties of measured densities increase [63, 66, 67], but will still be almost one magnitude smaller than the corresponding uncertainties achieved by a VTD. Low uncertainties are essential when developing fundamental equations of state or reference correlations for densities [69], as these models are limited to the accuracy of the underlying experimental datasets. However, when the lowest uncertainties are not of interest, e.g., for technical applications, the main advantage of precision densimeters diminishes and the utilization of fast and robust VTDs becomes feasible. Especially when liquids with high densities are considered, the low relative uncertainties, in the present study of less than approximately 0.10 %, are highly competitive. This is also the case when the fluids of interest are not available in high purity and the achievable combined uncertainties in density are constrained by the impurities of the samples.

### Complexity of Development and Operation

Although the lowest uncertainties are generally desirable, the measures required to achieve these are equally of concern. It can be assumed that the complexity of an experimental system increases over-proportionally with decreasing measurement uncertainty. This becomes clear when considering the development of both systems compared here. The development and commissioning of the sophisticated and custom-built single-sinker cryogenic densimeter took approximately 7 years from the conceptual design [65] to its reliable operation [64]. However, the commissioning of the relatively simple measuring system used in this work took about three months, including the planning phase, the assembly, which took about 2 weeks, and the calibration measurements. This comparably short time to operational readiness is enabled by the commercial availability of ready-to-use VTDs and the utilization of an environmental chamber instead of a vacuum-insulated cryostat, but also by the fast measurement workflow. While the investigation of an isotherm with the single-sinker densimeter takes two to three days in most cases, it was possible to investigate two isotherms in one day with the commissioned VTD setup. Therefore, with appropriate preparation, the VTD calibration as described in Sects. 4.1 and 4.2 can be carried out in 2 to 3 weeks. This measurement rate can also be a decisive advantage when ($p$, $\rho$, $T$) data are of interest for fluid systems that have been poorly examined or not at all. With a faster system, valuable data can be provided over a broad temperature and pressure range in a relatively short time frame. Another main advantage of the VTD is that the operation of the densimeter and the data analysis are significantly less comprehensive and can even be used in field applications. The system used in the present work can be operated after a short training period, while operating the complex gravimetric densimeter requires extensive briefing of the operators with subsequent elaborate processing of the recorded data (i.e., the correction of a force-transmission error [70, 71]).

### Investment and Operating Costs

It is apparent that the complexity of a system is also reflected in its cost. A precision apparatus like the single-sinker cryogenic densimeter is not only rather comprehensive, but the individual components themselves are correspondingly costly to be able to support the targeted measurement uncertainties. The hardware of the single-sinker densimeter as described in [60] can be valued at approximately €400 000 (before VAT), excluding the high personnel costs caused by the elaborate planning and development. VTDs, on the other hand, are available for less than €40 000 (before VAT). A system comparable to the experimental setup described in Sect. 2.1 can, therefore, be set up for less than €100 000 (before VAT), strongly depending on the model and configuration of the environmental chamber, which in turn can also be used for other applications. At the same time, the planning costs are also noticeably lower because less time is required.

In terms of operating costs, both systems will be about the same. Both systems utilize liquid nitrogen for cooling the core components. The supply of liquid nitrogen and the electric power consumption will be the key expenses, without considering personnel costs. Although the superior thermostat design of the densimeter results in lower nitrogen consumption, this advantage is offset by the increased time required. Thus, the liquid nitrogen demand per isotherm will be very similar to that of the VTD system. Regarding electricity, the single-sinker densimeter bears the higher consumption, just by utilizing an additional vacuum pump for the isolation vacuum and the number of required measuring instruments.

## Conclusions

This study reports the successful operation of a commercially available high-pressure vibrating tube densimeter at very low temperatures, down to 100 K. To evaluate the densimeter's performance beyond the manufacturer's specifications, it was calibrated at pressures up to 10.0 MPa and over a temperature range of (120 to 200) K. The calibration was carried out using two established models with measurements under vacuum and on research-grade methane and propane. It was then validated with density measurements on pure ethane and argon.

First measurements at temperatures below approximately 180 K revealed that the vacuum oscillation period steadily increased during dwell times at constant temperature and with thermal cycling. Although the observed drift diminished after multiple cycles, it never completely stopped. This persistent change in the VTD vacuum characteristic impaired the application of the calibration models, which use quadratic polynomials to describe the temperature dependence of the vacuum oscillation period. Accordingly, for the calibration presented in this work, experimental values of the vacuum oscillation period were recorded after every isotherm. These experimental values were then used instead of quadratic polynomials to determine the fluid densities, which entails considerably increased experimental effort but allows for substantially more reliable results.

The calibration measurements on methane and propane were each carried out at five isotherms from (120 to 200) K and five pressures from (2 to 10) MPa, resulting in 50 recorded ($p$, $T$, $\tau$) data points. The determination of experimental densities according to the modified calibration models by May *et al.* [49, 50] and Outcalt and McLinden [47] yielded deviations from the values calculated with the respective reference equations of state of ± 0.39 kg·m^−3^ and ± 0.10 kg·m^−3^, respectively. The calibration has been validated with measurements of ethane and argon, yielding deviations from the equations of state of less than ± 0.55 kg·m^−3^ for the modified May *et al.* [49, 50] model and in the range of (− 0.47 to 0.16) kg·m^−3^ for the modified Outcalt and McLinden [47] model. The estimated uncertainties for the ethane and argon measurements are less than 2.43 kg·m^−3^ and less than 1.22 kg·m^−3^, respectively. The results deteriorate noticeably when not using experimental values for the vacuum oscillation period but quadratic polynomials. In this case, the validation measurements of ethane and argon show deviations from the reference equations of state from (− 1.45 to 2.65) kg·m^−3^ when applying the original model by May *et al.* [49, 50] and deviations from (− 1.59 to 2.69) kg·m^−3^ for the model by Outcalt and McLinden [47].

In summary, the low-temperature study presented in this work demonstrated that the utilized densimeter can be operated at temperatures down to 100 K. Furthermore, the calibration carried out shows that the VTD is capable of reliable density measurements at temperatures of at least down to 120 K when the shifting vacuum characteristics are taken into account. This opens up new fields of application for VTDs, where they can be a feasible and accessible alternative to conventional measuring methods. As an outlook, future works may investigate whether the shift of the vacuum oscillation period will come to a standstill with a long-term study by constructive modifications at the densimeter unit or through an alternative operating protocol that avoids measurements of the vacuum oscillations period. Moreover, a worthwhile study would demonstrate whether a VTD-based apparatus can be used for density measurements on liquefied gas mixtures without changes in the composition of the liquefied sample [72, 73].

### Supplementary Information

Below is the link to the electronic supplementary material.

(PDF 317 kb)

## Data Availability

All data required to understand the present work are provided in the Supplementary Information.
