# Supplementary material for: Utilization of a High-Pressure Vibrating Tube Densimeter for Liquids at Temperatures Down to 100 K
Source: Int J Thermophys. 2024 Apr 19;45(5):72. doi: 10.1007/s10765-024-03357-9 (PMC11031472; doi:10.1007/s10765-024-03357-9)
Supplement: Supplementary file 1 — (PDF 317 kb) [file 10765_2024_3357_MOESM1_ESM.pdf]

International Journal of Thermophysics – Electronic Supplementary Material to the Paper

Utilization of a High-Pressure Vibrating-Tube Densimeter for Liquids at Temperatures Down to 100 K

Nils von Preetzmann[1], Daniel Zipplies[2], Roland Span[1], and Markus Richter[2,*],

[1] Faculty of Mechanical Engineering, Thermodynamics, Ruhr University Bochum, Germany

[2] Department of Mechanical Engineering, Applied Thermodynamics,

Chemnitz University of Technology, Germany

* Corresponding author. Tel.: +49-371-531-38050, E-mail: m.richter@mb.tu-chemnitz.de

ORCID IDs: 0000-0003-2523-3743, 0000-0002-8350-8285, 0000-0001-8120-5646

Electronic Supplementary Material - Online Resource:

As mentioned in Section 4.1 of the paper, the vacuum oscillation period of the DMA HPM vibrating-tube densimeter exhibited an increase during dwell times and with thermal cycling at temperatures below 180 K. Hence, as described in Section 4.2, two sets of calibration parameters for both models of May et al. [1,2] and Outcalt and McLinden [3] were determined (listed in Table 2 of the paper). One set where the vacuum characteristics were described with quadratic polynomials $\tau_0(T)$, as recommended by the authors of the calibration models, and one set of parameters where experimental values $\tau_{0,\mathrm{exp}}$ were used. The results for the calibration measurements on methane and propane when using experimental vacuum oscillation periods $\tau_{0,\mathrm{exp}}$ are given in Section S1, Table S1 and Table S2, listing the experimental densities and their deviations from the corresponding reference equations of state. For both calibration models, densities were determined using the corresponding calibration parameters listed in Table 2 of the paper. The results of the validation measurements on ethane and argon are given in Table 3 of the paper. Their estimated experimental uncertainties are listed in Section S3. The results for the calibration and validation measurements when applying the quadratic polynomials are given in Section S2.

S1 Experimental Results of the Calibration Measurements (to Section 4.2)

The experimental results reported in this section refer to the modified calibration models where experimental vacuum oscillation periods $\tau_{0,\mathrm{exp}}$ are used instead of the quadratic polynomials that are proposed by the authors of the calibration models [1–3]. The calibration parameters for the determination of $A(p,T)$ and $B(p,T)$ according to both models are given in Table 2 of the paper.

Table S1 Results of the calibration measurements of methane for experimentally determined vacuum oscillation periods $\tau_{0,\mathrm{exp}}$, where T is the temperature, p is the pressure, and τ is the oscillation period. ρ_{EOS} is the density calculated with the reference equations of state by Setzmann and Wagner [4], ρ_{May} is the density calculated with the modified May et al. [1,2] model, and ρ_{OM} is the density calculated with the modified Outcalt and McLinden [3] model. $\Delta\rho_i$ are the corresponding deviations from ρ_{EOS}

T/K	p/MPa	τ/μs	ρ_{EOS} /kg·m^{-3}	ρ_{May}[a] /kg·m^{-3}	$\Delta\rho_{\mathrm{May}}$ /kg·m^{-3}	ρ_{OM}[a] /kg·m^{-3}	$\Delta\rho_{\mathrm{OM}}$ /kg·m^{-3}
200.02	10.008	2578.546	266.20	266.46	0.26	266.15	−0.05
200.01	8.001	2576.761	242.74	242.93	0.19	242.75	0.01
200.00	6.093	2571.451	172.79	172.79	0.00	172.69	−0.10
200.00	3.973	2562.606	56.09	56.14	0.05	56.14	0.05
200.00	1.956	2559.991	21.82	21.72	−0.10	21.83	0.01
180.05	10.064	2576.547	319.70	319.94	0.24	319.74	0.04
180.04	7.958	2575.881	311.06	311.21	0.15	311.10	0.04
180.04	6.046	2575.106	300.96	301.03	0.07	300.97	0.01
180.04	3.917	2573.814	284.02	283.97	−0.05	283.97	−0.05
180.04	1.962	2554.360	26.22	26.21	−0.01	26.32	0.10
160.02	10.001	2573.609	358.77	358.73	−0.04	358.80	0.03
160.01	7.996	2573.268	354.47	354.32	−0.15	354.49	0.02
160.00	5.936	2572.879	349.53	349.27	−0.26	349.52	−0.01
159.99	3.932	2572.450	344.03	343.68	−0.35	343.98	−0.05
159.99	1.986	2571.968	337.77	337.38	−0.39	337.69	−0.08
139.92	10.050	2570.371	391.40	391.33	−0.07	391.39	−0.01
139.92	8.088	2570.159	388.76	388.64	−0.12	388.74	−0.02
139.92	6.022	2569.924	385.81	385.66	−0.15	385.78	−0.03
139.91	3.973	2569.676	382.67	382.49	−0.18	382.62	−0.05
139.91	1.986	2569.418	379.40	379.18	−0.22	379.31	−0.09
119.89	10.005	2567.055	419.94	419.92	−0.02	419.94	0.00
119.88	7.916	2566.897	418.01	417.97	−0.04	418.03	0.02
119.87	5.979	2566.747	416.15	416.12	−0.03	416.21	0.06
119.87	4.015	2566.589	414.19	414.16	−0.03	414.24	0.05
119.87	1.971	2566.417	412.05	412.01	−0.04	412.08	0.03

[fa]Due to temperature deviations between the fluid and vacuum measurements, the values of $\tau_{0,\mathrm{exp}}$ were corrected with $\tau_0' = \tau_{0,\mathrm{exp}} + \left(T - T_{0,\mathrm{exp}}\right) \cdot \partial\tau_0/\partial T$. The sensitivity $\partial\tau_0/\partial T$ is estimated with the temperature-derivative of the respective quadratic fit $\tau_{0,\mathrm{fit}}(T)$ as given in Table 2 of the paper

Table S2 Results of the calibration measurements of propane for experimentally determined vacuum oscillation periods $\tau_{0,\mathrm{exp}}$, where T is the temperature, p is the pressure, and τ is the oscillation period. ρ_{EOS} is the density calculated with the reference equations of state by Lemmon et al. [5], ρ_{May} is the density calculated with the modified May et al. [1,2] model, and ρ_{OM} is the density calculated with the modified Outcalt and McLinden [3] model. $\Delta\rho_i$ are the corresponding deviations from ρ_{EOS}

T/K	p/MPa	τ/μs	ρ_{EOS} /kg·m^{-3}	ρ_{May}[a] /kg·m^{-3}	$\Delta\rho_{\mathrm{May}}$ /kg·m^{-3}	ρ_{OM}[a] /kg·m^{-3}	$\Delta\rho_{\mathrm{OM}}$ /kg·m^{-3}
200.05	10.008	2605.233	623.14	623.36	0.22	623.15	0.01
200.02	8.001	2605.096	621.68	621.77	0.09	621.70	0.02
199.97	6.016	2604.954	620.23	620.11	−0.12	620.26	0.03
199.97	4.006	2604.822	618.66	618.59	−0.07	618.69	0.03
199.97	2.001	2604.685	617.07	617.00	−0.07	617.09	0.02
180.01	10.007	2600.607	643.09	643.10	0.01	643.06	−0.03
180.01	8.002	2600.495	641.84	641.85	0.01	641.80	−0.04
180.01	6.003	2600.383	640.57	640.60	0.03	640.54	−0.03
180.01	4.001	2600.268	639.27	639.31	0.04	639.22	−0.05
180.01	1.997	2600.153	637.95	638.01	0.06	637.88	−0.07
159.99	9.998	2596.136	662.73	662.72	−0.01	662.78	0.05
159.99	8.001	2596.040	661.68	661.69	0.01	661.74	0.06
159.99	6.001	2595.942	660.60	660.63	0.03	660.65	0.05
159.99	3.996	2595.843	659.51	659.55	0.04	659.53	0.02
159.99	1.998	2595.746	658.41	658.50	0.09	658.42	0.01
139.92	10.001	2591.869	682.29	682.06	−0.23	682.25	−0.04
139.92	8.000	2591.788	681.39	681.23	−0.16	681.39	0.00
139.92	5.993	2591.703	680.48	680.35	−0.13	680.48	0.00
139.91	3.993	2591.619	679.56	679.48	−0.08	679.57	0.01
139.91	1.997	2591.535	678.62	678.61	−0.01	678.62	0.00
119.86	9.994	2587.937	701.89	701.90	0.01	701.83	−0.06
119.86	8.006	2587.865	701.12	701.20	0.08	701.08	−0.04
119.86	6.005	2587.795	700.33	700.52	0.19	700.34	0.01
119.86	4.000	2587.726	699.54	699.86	0.32	699.60	0.06
119.86	1.997	2587.652	698.74	699.13	0.39	698.77	0.03

[a] Due to temperature deviations between the fluid and vacuum measurements, the values of $\tau_{0,\mathrm{exp}}$ were corrected with $\tau_0' = \tau_{0,\mathrm{exp}} + \left(T - T_{0,\mathrm{exp}}\right) \cdot \partial\tau_0/\partial T$. The sensitivity $\partial\tau_0/\partial T$ is estimated with the temperature-derivative of the respective quadratic fit $\tau_{0,\mathrm{fit}}(T)$ as given in Table 2 of the paper

S2 Results for $\tau_0(T)$ Calculated with Quadratic Polynomials (to Section 4.2)

The experimental results reported in this section refer to the densities determined by using vacuum oscillation periods calculated with quadratic polynomials $\tau_{0,\mathrm{fit}}(T)$. The calibration parameters for the determination of $A(p,T)$ and $B(p,T)$ according to both models [1–3] are given in Table 2 of the paper.

Table S3 Results of the calibration measurements of methane when using the quadratic polynomials $\tau_{0,\mathrm{fit}}(T)$, where T is the temperature, p is the pressure, and τ is the oscillation period. ρ_{EOS} is the density calculated with the reference equations of state by Setzmann and Wagner [4], ρ_{May} is the density calculated with the model by May et al. [1,2], and ρ_{OM} is the density calculated with the model by Outcalt and McLinden [3]. $\Delta\rho_i$ are the corresponding deviations from ρ_{EOS}

T/K	p/MPa	τ/μs	ρ_{EOS} /kg·m^{-3}	ρ_{May} /kg·m^{-3}	$\Delta\rho_{\mathrm{May}}$ /kg·m^{-3}	ρ_{OM} /kg·m^{-3}	$\Delta\rho_{\mathrm{OM}}$ /kg·m^{-3}
200.02	10.008	2578.546	266.20	266.07	−0.12	266.20	0.00
200.01	8.001	2576.761	242.74	242.77	0.02	242.81	0.07
200.00	6.093	2571.451	172.79	172.67	−0.12	172.67	−0.12
200.00	3.973	2562.606	56.09	56.10	0.01	56.10	0.01
200.00	1.956	2559.991	21.82	22.13	0.32	22.19	0.37
180.05	10.064	2576.547	319.70	319.90	0.21	319.60	−0.10
180.04	7.958	2575.881	311.06	311.41	0.35	310.97	−0.09
180.04	6.046	2575.106	300.96	301.44	0.47	300.90	−0.06
180.04	3.917	2573.814	284.02	284.62	0.59	284.01	−0.01
180.04	1.962	2554.360	26.22	26.70	0.48	25.60	−0.62
160.02	10.001	2573.609	358.77	358.75	−0.02	358.83	0.06
160.01	7.996	2573.268	354.47	354.58	0.11	354.51	0.04
160.00	5.936	2572.879	349.53	349.77	0.24	349.59	0.06
159.99	3.932	2572.450	344.03	344.41	0.37	344.13	0.10
159.99	1.986	2571.968	337.77	338.32	0.55	337.99	0.22
139.92	10.050	2570.371	391.40	391.05	−0.35	391.63	0.23
139.92	8.088	2570.159	388.76	388.53	−0.23	388.95	0.19
139.92	6.022	2569.924	385.81	385.73	−0.08	386.01	0.20
139.91	3.973	2569.676	382.67	382.75	0.08	382.92	0.25
139.91	1.986	2569.418	379.40	379.63	0.23	379.71	0.31
119.89	10.005	2567.055	419.94	419.24	−0.71	419.77	−0.17
119.88	7.916	2566.897	418.01	417.47	−0.54	417.82	−0.19
119.87	5.979	2566.747	416.15	415.79	−0.37	415.99	−0.16
119.87	4.015	2566.589	414.19	413.98	−0.21	414.05	−0.14
119.87	1.971	2566.417	412.05	412.00	−0.05	411.96	−0.09

Table S4 Results of the calibration measurements of propane when using the quadratic polynomials $\tau_{0,\text{fit}}(T)$, where T is the temperature, p is the pressure, and τ is the oscillation period. ρ_{EOS} is the density calculated with the reference equations of state by Lemmon et al. [5], ρ_{May} is the density calculated with the model by May et al. [1,2], and ρ_{OM} is the density calculated with the model by Outcalt and McLinden [3]. $\Delta\rho_i$ are the corresponding deviations from ρ_{EOS}

T/K	p/MPa	τ/μs	ρ_{EOS} /kg·m^{-3}	ρ_{May} /kg·m^{-3}	$\Delta\rho_{\text{May}}$ /kg·m^{-3}	ρ_{OM} /kg·m^{-3}	$\Delta\rho_{\text{OM}}$ /kg·m^{-3}
200.05	10.008	2605.233	623.14	623.29	0.15	623.07	−0.07
200.02	8.001	2605.096	621.68	621.76	0.08	621.62	−0.06
199.97	6.016	2604.954	620.23	620.26	0.03	620.18	−0.05
199.97	4.006	2604.822	618.66	618.64	−0.02	618.62	−0.04
199.97	2.001	2604.685	617.07	617.02	−0.05	617.04	−0.03
180.01	10.007	2600.607	643.09	643.37	0.28	643.24	0.15
180.01	8.002	2600.495	641.84	642.04	0.20	641.98	0.14
180.01	6.003	2600.383	640.57	640.71	0.14	640.70	0.13
180.01	4.001	2600.268	639.27	639.34	0.07	639.37	0.10
180.01	1.997	2600.153	637.95	637.97	0.02	638.03	0.08
159.99	9.998	2596.136	662.73	662.58	−0.15	662.77	0.04
159.99	8.001	2596.040	661.68	661.46	−0.22	661.71	0.03
159.99	6.001	2595.942	660.60	660.30	−0.30	660.60	0.00
159.99	3.996	2595.843	659.51	659.13	−0.38	659.46	−0.05
159.99	1.998	2595.746	658.41	657.99	−0.42	658.32	−0.09
139.92	10.001	2591.869	682.29	681.82	−0.47	682.02	−0.27
139.92	8.000	2591.788	681.39	680.87	−0.52	681.14	−0.25
139.92	5.993	2591.703	680.48	679.90	−0.58	680.21	−0.27
139.91	3.993	2591.619	679.56	678.93	−0.63	679.26	−0.30
139.91	1.997	2591.535	678.62	677.95	−0.67	678.29	−0.33
119.86	9.994	2587.937	701.89	702.65	0.76	702.04	0.15
119.86	8.006	2587.865	701.12	701.81	0.69	701.28	0.16
119.86	6.005	2587.795	700.33	701.00	0.67	700.51	0.18
119.86	4.000	2587.726	699.54	700.20	0.66	699.74	0.20
119.86	1.997	2587.652	698.74	699.34	0.60	698.87	0.13

Table S5 Results of the validation measurements of ethane when using the quadratic polynomials $\tau_{0,\mathrm{fit}}(T)$, where T is the temperature, p is the pressure, and τ is the oscillation period. ρ_{EOS} is the density calculated with the reference equations of state by Bücker and Wagner [6], ρ_{May} is the density calculated with the model by May et al. [1,2], and ρ_{OM} is the density calculated with the model by Outcalt and McLinden [3]. $\Delta\rho_i$ are the corresponding deviations from ρ_{EOS}

T/K	p/MPa	τ/μs	ρ_{EOS} /kg·m^{-3}	ρ_{May} /kg·m^{-3}	$\Delta\rho_{\mathrm{May}}$ /kg·m^{-3}	ρ_{OM} /kg·m^{-3}	$\Delta\rho_{\mathrm{OM}}$ /kg·m^{-3}
199.99	10.003	2598.525	534.81	533.37	−1.44	533.23	−1.58
199.99	8.008	2598.356	532.76	531.33	−1.43	531.23	−1.53
199.99	6.000	2598.181	530.62	529.23	−1.39	529.17	−1.45
199.99	4.001	2597.997	528.41	527.01	−1.40	526.99	−1.42
199.99	2.016	2597.812	526.13	524.77	−1.36	524.80	−1.33
179.95	10.006	2594.202	558.14	557.28	−0.86	557.10	−1.04
179.93	7.996	2594.057	556.52	555.61	−0.91	555.45	−1.07
179.93	5.999	2593.913	554.85	553.93	−0.92	553.79	−1.06
179.92	3.999	2593.768	553.13	552.22	−0.91	552.09	−1.04
179.92	2.000	2593.618	551.36	550.45	−0.91	550.34	−1.02
160.01	9.990	2590.145	580.43	581.49	1.06	581.65	1.22
160.00	7.996	2590.030	579.12	580.18	1.06	580.34	1.22
159.99	5.991	2589.913	577.77	578.84	1.07	579.01	1.24
159.98	4.000	2589.793	576.40	577.48	1.08	577.65	1.25
159.98	2.075	2589.678	575.05	576.15	1.10	576.31	1.26
139.93	10.003	2586.055	602.29	602.93	0.64	603.23	0.94
139.92	7.987	2585.959	601.19	601.85	0.66	602.16	0.97
139.92	5.995	2585.866	600.10	600.82	0.72	601.12	1.02
139.92	3.991	2585.772	598.97	599.75	0.78	600.04	1.07
139.91	2.001	2585.678	597.84	598.69	0.85	598.96	1.12
119.99	9.999	2582.305	623.60	625.54	1.94	625.24	1.64
119.99	7.998	2582.225	622.70	624.65	1.95	624.36	1.66
119.98	5.994	2582.144	621.79	623.77	1.98	623.47	1.68
119.98	3.910	2582.059	620.82	622.82	2.00	622.50	1.68
119.97	2.026	2581.981	619.95	621.97	2.02	621.62	1.67
169.96	10.006	2592.311	569.40	571.65	2.25	571.63	2.23
169.95	9.005	2592.244	568.69	570.92	2.23	570.91	2.22
169.93	7.005	2592.116	567.22	569.47	2.25	569.47	2.25
169.93	5.003	2591.987	565.70	567.98	2.28	567.99	2.29
169.92	3.276	2591.873	564.37	566.65	2.28	566.67	2.30

Table S6 Results of the validation measurements of argon when using the quadratic polynomials $\tau_{0,\mathrm{fit}}(T)$, where T is the temperature, p is the pressure, and τ is the oscillation period. ρ_{EOS} is the density calculated with the reference equations of state by Tegeler et al. [7], ρ_{May} is the density calculated with the model by May et al. [1,2], and ρ_{OM} is the density calculated with the model by Outcalt and McLinden [3]. $\Delta\rho_i$ are the corresponding deviations from ρ_{EOS}

T/K	p/MPa	τ/μs	ρ_{EOS} /kg·m^{-3}	ρ_{May} /kg·m^{-3}	$\Delta\rho_{\mathrm{May}}$ /kg·m^{-3}	ρ_{OM} /kg·m^{-3}	$\Delta\rho_{\mathrm{OM}}$ /kg·m^{-3}
169.96	10.013	2598.931	658.43	661.08	2.65	661.12	2.69
169.95	9.005	2592.703	574.78	577.09	2.31	577.09	2.31
169.94	7.985	2584.300	462.02	464.11	2.09	463.97	1.95
169.94	6.710	2573.996	324.38	326.16	1.78	325.77	1.39
169.94	5.990	2569.453	263.93	265.57	1.64	265.05	1.12
169.94	4.756	2563.425	183.86	185.45	1.59	184.71	0.85

S3 Estimated Uncertainties in Density (to Section 4.3)

In Section 4.3 of the manuscript, an uncertainty analysis has been presented. The uncertainty budget is determined in line with the "Guide to the Expression of Uncertainty in Measurement" [8] (ISO/IEC Guide), referred to as GUM. The combined expanded uncertainty in density $U(\rho)$ is determined by Equation (10) in the manuscript, covering contributions of the measurements uncertainties of pressure $u(p)$, temperature $u(T)$ and oscillation period $u(\tau)$, as well as the uncertainties resulting from the reproducibility $u(\rho_{\mathrm{repro}})$, calibration $u(\rho_{\mathrm{cal}})$, reference equations of state $u(\rho_{\mathrm{EOS}})$, and the temperature correction of the vacuum oscillation period $u(\tau_{0,\mathrm{corr}})$.

Here, the contribution of the calibration $u(\rho_{\mathrm{cal}})$ varies with the used calibration model. It has been conservatively defined as the root of the sum of squared residuals between the experimental densities and the densities calculated with the reference equations of state. Using measured values for the vacuum oscillation period, this contribution is 1.095 kg·m^{-3} for the modified May et al model and 0.306 kg·m^{-3} for the modified Outcalt and McLinden model. The resulting individual uncertainties for all (p, ρ, T) state points of the validation measurements are listed in Table S7 and Table S8.

Table S7 Experimental uncertainties of the validation measurements of ethane for experimentally determined vacuum oscillation periods $\tau_{0,\mathrm{exp}}$, where T is the temperature, p is the pressure, and ρ_{EOS} is the density calculated with the reference equations of state by Bücker and Wagner [6] for ethane, ρ_{May} is the experimental density calculated with the modified May et al. [1,2] model, and ρ_{OM} is the experimental density calculated with the modified Outcalt and McLinden [3] model. $U(\rho_{\mathrm{May}})$ and $U(\rho_{\mathrm{OM}})$ are the estimated expanded combined uncertainties (k = 2) for the corresponding models

T/K	p/MPa	ρ_{EOS} /kg·m^{-3}	ρ_{May}[a] /kg·m^{-3}	$U(\rho_{\mathrm{May}})$ /kg·m^{-3}	ρ_{OM}[a] /kg·m^{-3}	$U(\rho_{\mathrm{OM}})$ /kg·m^{-3}
199.99	10.003	534.81	535.19	1.60	534.87	0.52
199.99	8.008	532.76	533.15	1.60	532.85	0.52
199.99	6.000	530.62	531.02	1.60	530.74	0.52
199.99	4.001	528.41	528.77	1.60	528.50	0.52
199.99	2.016	526.13	526.51	1.60	526.23	0.52
179.95	10.006	558.14	558.47	1.60	558.30	0.53
179.93	7.996	556.52	556.75	1.60	556.66	0.52
179.93	5.999	554.85	555.04	1.60	554.97	0.52
179.92	3.999	553.13	553.32	1.60	553.25	0.52
179.92	2.000	551.36	551.53	1.60	551.44	0.52
160.01	9.990	580.43	580.31	1.60	580.52	0.53
160.00	7.996	579.12	578.99	1.60	579.23	0.53
159.99	5.991	577.77	577.65	1.60	577.89	0.53
159.98	4.000	576.40	576.26	1.60	576.52	0.53
159.98	2.075	575.05	574.94	1.60	575.16	0.53
139.93	10.003	602.29	602.15	1.60	602.19	0.52
139.92	7.987	601.19	601.09	1.60	601.14	0.52
139.92	5.995	600.10	600.08	1.60	600.11	0.52
139.92	3.991	598.97	599.05	1.60	599.04	0.52
139.91	2.001	597.84	598.01	1.60	597.95	0.52
119.99	9.999	623.60	623.18	1.60	623.22	0.53
119.99	7.998	622.70	622.35	1.60	622.37	0.53
119.98	5.994	621.79	621.50	1.60	621.50	0.53
119.98	3.910	620.82	620.60	1.60	620.54	0.53
119.97	2.026	619.95	619.78	1.60	619.67	0.53
169.96	10.006	569.40	569.11	1.60	569.31	0.53
169.95	9.005	568.69	568.32	1.60	568.60	0.53
169.93	7.005	567.22	566.83	1.60	567.15	0.53
169.93	5.003	565.70	565.32	1.60	565.66	0.53
169.92	3.276	564.37	563.99	1.60	564.31	0.53

[a] Due to temperature deviations between the fluid and vacuum measurements, the values of $\tau_{0,\mathrm{exp}}$ were corrected with $\tau_0' = \tau_{0,\mathrm{exp}} + (T - T_{0,\mathrm{exp}}) \cdot \partial\tau_0/\partial T$. The sensitivity $\partial\tau_0/\partial T$ is determined approximately with the derivative of the respective quadratic fit $\tau_{0,\mathrm{fit}}(T)$

Table S8 Experimental uncertainties of the validation measurements of argon for experimentally determined vacuum oscillation periods $\tau_{0,\mathrm{exp}}$, where T is the temperature, p is the pressure, and ρ_{EOS} is the density calculated with the reference equations of state by Tegeler et al. [7], ρ_{May} is the experimental density calculated with the modified May et al. [1,2] model and ρ_{OM} is the experimental density calculated with the modified Outcalt and McLinden [3] model. $U(\rho_{\mathrm{May}})$ and $U(\rho_{\mathrm{OM}})$ are the estimated expanded combined uncertainties (k = 2) for the corresponding models

T/K	p/MPa	ρ_{EOS} /kg·m^{-3}	ρ_{May}[a] /kg·m^{-3}	$U(\rho_{\mathrm{May}})$ /kg·m^{-3}	ρ_{OM}[a] /kg·m^{-3}	$U(\rho_{\mathrm{OM}})$ /kg·m^{-3}
169.96	10.013	658.43	657.88	1.79	657.96	0.95
169.95	9.005	574.78	574.34	1.88	574.42	1.11
169.94	7.985	462.02	461.86	1.89	461.93	1.13
169.94	6.710	324.38	324.37	1.74	324.46	0.86
169.94	5.990	263.93	263.92	1.69	264.02	0.75
169.94	4.756	183.86	183.85	1.64	183.99	0.64

[a] Due to temperature deviations between the fluid and vacuum measurements, the values of $\tau_{0.\mathrm{exp}}$ were corrected with $\tau_0' = \tau_{0,\mathrm{exp}} + (T - T_{0,\mathrm{exp}}) \cdot \partial\tau_0/\partial T$. The sensitivity $\partial\tau_0/\partial T$ is determined approximately with the derivative of the respective quadratic fit $\tau_{0,\mathrm{fit}}(T)$

References

[1] E.F. May, W.J. Tay, M. Nania, A. Aleji, S.Z. Al Ghafri, J.P.M. Trusler, Physical apparatus parameters and model for vibrating tube densimeters at pressures to 140 MPa and temperatures to 473 K, Rev. Sci. Instrum. 85 (2014) 95111. https://doi.org/10.1063/1.4894469.

[2] E.F. May, W.J. Tay, M. Nania, A. Aleji, S.Z. Al Ghafri, J.P.M. Trusler, Erratum: "Physical apparatus parameters and model for vibrating tube densimeters at pressures to 140 MPa and temperatures to 473 K" Rev. Sci. Instrum. 85, 095111 (2014), Rev. Sci. Instrum. 86 (2015) 49902. https://doi.org/10.1063/1.4919437.

[3] S.L. Outcalt, M.O. McLinden, Automated Densimeter for the Rapid Characterization of Industrial Fluids, Ind. Eng. Chem. Res. 46 (2007) 8264–8269. https://doi.org/10.1021/ie070791e.

[4] U. Setzmann, W. Wagner, A New Equation of State and Tables of Thermodynamic Properties for Methane Covering the Range from the Melting Line to 625 K at Pressures up to 100 MPa, J. Phys. Chem. Ref. Data 20 (1991) 1061–1155. https://doi.org/10.1063/1.555898.

[5] E.W. Lemmon, M.O. McLinden, W. Wagner, Thermodynamic Properties of Propane. III. A Reference Equation of State for Temperatures from the Melting Line to 650 K and Pressures up to 1000 MPa, J. Chem. Eng. Data 54 (2009) 3141–3180. https://doi.org/10.1021/je900217v.

[6] D. Bücker, W. Wagner, A Reference Equation of State for the Thermodynamic Properties of Ethane for Temperatures from the Melting Line to 675 K and Pressures up to 900 MPa, J. Phys. Chem. Ref. Data 35 (2006) 205–266. https://doi.org/10.1063/1.1859286.

[7] C. Tegeler, R. Span, W. Wagner, A New Equation of State for Argon Covering the Fluid Region for Temperatures From the Melting Line to 700 K at Pressures up to 1000 MPa, J. Phys. Chem. Ref. Data 28 (1999) 779–850. https://doi.org/10.1063/1.556037.

[8] International Organization for Standardization, ISO/IEC Guide 98-3:2008, Uncertainty of measurement – Part 3: Guide to the expression of uncertainty in measurement, Geneva, 2008.
